# Murine Gammaherpesvirus M2 Protein Induction of IRF4 via the NFAT Pathway Leads to IL-10 Expression in B Cells

**DOI:** 10.1371/journal.ppat.1003858

**Published:** 2014-01-02

**Authors:** Udaya S. Rangaswamy, Samuel H. Speck

**Affiliations:** 1 Microbiology and Molecular Genetics Graduate Program, Emory University School of Medicine, Atlanta, Georgia, United States of America; 2 Department of Microbiology & Immunology, Emory University School of Medicine, Atlanta, Georgia, United States of America; 3 Emory Vaccine Center, Emory University School of Medicine, Atlanta, Georgia, United States of America; Medical College of Wisconsin, United States of America

## Abstract

Reactivation of the gammaherpesviruses Epstein-Barr virus (EBV), Kaposi's sarcoma-associated herpesvirus (KSHV) and murine gammaherpesvirus 68 (MHV68) from latently infected B cells has been linked to plasma cell differentiation. We have previously shown that the MHV68 M2 protein is important for virus reactivation from B cells and, when expressed alone in primary murine B cells, can drive B cell differentiation towards a pre-plasma cell phenotype. In addition, expression of M2 in primary murine B cells leads to secretion of high levels of IL-10 along with enhanced proliferation and survival. Furthermore, the absence of M2 *in vivo* leads to a defect in the appearance of MHV68 infected plasma cells in the spleen at the peak of MHV68 latency. Here, employing an inducible B cell expression system, we have determined that M2 activates the NFAT pathway in a Src kinase-dependent manner – leading to induction of the plasma cell-associated transcription factor, Interferon Regulatory Factor-4 (IRF4). Furthermore, we show that expression of IRF4 alone in a B cell line up-regulates IL-10 expression in culture supernatants, revealing a novel role for IRF4 in B cell induced IL-10. Consistent with the latter observation, we show that IRF4 can regulate the IL-10 promoter in B cells. In primary murine B cells, addition of cyclosporine (CsA) resulted in a significant decrease in M2-induced IL-10 levels as well as IRF4 expression, emphasizing the importance of the NFAT pathway in M2- mediated induction of IL-10. Together, these studies argue in favor of a model wherein M2 activation of the NFAT pathway initiates events leading to increased levels of IRF4 – a key player in plasma cell differentiation – which in turn triggers IL-10 expression. In the context of previous findings, the data presented here provides insights into how M2 facilitates plasma cell differentiation and subsequent virus reactivation.

## Author Summary

The human viruses Epstein-Barr Virus (EBV) and Kaposi's Sarcoma-associated herpesvirus (KSHV) are members of the gammaherpesvirus family – pathogens that are associated with cancers of lymphoid tissues. Murine gammaherpesvirus 68 (MHV68) infection of laboratory mice provides a small animal model to study how this family of viruses chronically infects their host. The gammaherpesvirus establish a quiescent infection (termed latency) for the lifetime of the individual. However, they are capable of producing progeny virus (termed reactivation) in response to a variety of immune or environmental stimuli. Differentiation of latently infected B cells into plasma cells (the cells producing antibodies) has been associated with reactivation from latency. Notably, the MHV68 M2 protein plays a role in driving differentiation of MHV68 infected B cells to plasma cells. Furthermore, M2 expression results in increased levels of IL-10 (an immune-regulatory cytokine). Here we show that M2 mediated IL-10 production occurs through induction of IRF4 expression, a key player in plasma cell differentiation. This process involves Src kinases and NFAT – both components of B cell receptor signaling. Additionally, mice lacking IRF4 in infected cells show a significant defect in virus reactivation, thereby identifying IRF4 as a crucial component of M2 mediated functions.

## Introduction

Gammaherpesviruses are lymphotropic viruses that are associated with the development of lymphoproliferative diseases and lymphomas (reviewed in [1]). The two human γ-herpesviruses, Epstein Barr virus (EBV) and Kaposi's sarcoma-associated herpesvirus (KSHV), exhibit very narrow host tropism, making *in vivo* studies challenging and accentuating the need for relevant small animal models. One of the best-characterized animal models of gammaherpesvirus pathogenesis is murine gammaherpesvirus 68 (MHV68) infection of laboratory strains of mice [2], [3]. Intranasal inoculation of mice with MHV68 results in an acute lytic phase in the lung that is largely cleared by days 10–12 post infection. Latency is established in the spleen and, similar to EBV, memory B cells are the primary long-term latency reservoir. Periodic reactivation results in productive infectious virus capable of reseeding new latency reservoirs. Efficient trafficking of MHV68 to the spleen requires B cells, as evidenced by the delayed kinetics of establishment of splenic latency in B cell-deficient mice [4]–[7].

Both EBV and KSHV encode genes that modulate the host B cell signaling to gain access to the latency reservoir. EBV encodes LMP1 and LMP2a, which mimic signals from CD40 and BCR respectively, and vIL-10- a homolog of cellular IL-10, capable of inducing B cell proliferation [8]–[10]. Similarly, KSHV encodes K1, a constitutive BCR mimic that functions to activate the PI3K and NFAT signaling pathways [11], [12]. MHV68 does not encode clear homologs of these viral proteins, but the functions of the latency associated M2 gene product closely resemble those of EBV LMP2a and KSHV K1. The M2 ORF is not required for acute replication in the lung, but it is essential (in a dose and route dependent manner) for efficient establishment of latency in the spleen. Furthermore, under conditions where M2 null MHV68 mutants establish latency, a significant defect in reactivation from B cells is observed [13], [14]. Consistent with this finding, M2 is required for efficient differentiation of MHV68 infected B cells to plasma cells *in vivo*, and M2 over-expression can drive plasma cell differentiation *in vitro* in a B lymphoma cell line [15]. Furthermore, M2 expression in primary murine B cells leads to proliferation, IL-10 expression and differentiation to a pre-plasma, memory B cell phenotype [16]. Although M2 is not homologous to any cellular or viral protein, it contains two tyrosines and nine PxxP motifs that can bind SH2 and SH3 containing cellular proteins respectively [17]. Indeed, Tyr^120^ and Tyr^129^ of M2 can bind the SH2 domains of Fyn and Vav1 forming a trimolecular complex, resulting in the phosphorylation of Vav1 by Fyn [18]. Moreover, *in vivo* analyses have shown that Tyr^120^ and Tyr^129^ are required for efficient reactivation from latency in splenocytes [17]. M2 has also recently been shown to interact with, and drive phosphorylation of PLCγ2, as well as inhibit downstream Akt pathway activation [19]. While M2 forms multiprotein complexes, detailed analyses of the functional consequences associated with such interactions and downstream signaling events is lacking.

Crosslinking of the B cell receptor (BCR) follows a highly coordinated cascade of intracellular events resulting in the formation of a microsignalosome, primarily consisting of Lyn (a Src family kinase), Syk, BLNK, PLCγ2, PI3K and Vav ([20], [21] and reviewed in [22] and [23]). Activation of PLCγ2 results in the hydrolysis of PtdIns-4, 5-P2 to IP3 and DAG. Binding of IP3 to its receptor on the endoplasmic reticulum (ER) membrane leads to release of Ca^2+^ from the ER stores. Upon depletion of the ER Ca^2+^ stores, an influx of extracellular calcium occurs, termed Store Operated Calcium (SOC) flux leading to activation of the calcineurin-NFAT pathway. The NFAT (Nuclear Factor of Activated T cells) family of transcription factors is a key player in the regulation of gene transcription in response to a variety of immune signals. Originally identified as a T-cell specific factor required for the transcription of the IL-2 promoter, the five different family members – NFATc1 (also known as NFAT2), NFATc2 (also known as NFAT1), NFATc3 (also known as NFAT4), NFATc4 (also known as NFAT3) and NFAT5 are widely expressed in most cell types and act in response to calcium mobilization (with the exception of NFAT5) (reviewed in [24]). The role of NFAT family members in B cells has only recently been appreciated. *In vivo* ablation of NFATc1 results in defective B1a B cell development [25]. Interestingly, mice lacking the regulatory subunit of calcineurin (Cnb1) in B cells exhibit impaired class switching by T-independent type II antigens and defective proliferation upon BCR engagement. These mice have a defect in plasma cell responses (potentially due to lack of sufficient IRF4 expression by these mice) [26], which is reminiscent of a phenotype of M2 null MHV68.

Interferon Regulatory Factor 4 (IRF4), a member of the IRF family of transcription factors, is expressed upon activation of T and B cells. In B cells, IRF4 is expressed in all stages of B cell development except germinal center B cells, and is highly expressed in terminally differentiated plasma cells (reviewed in [27]). Notably, IRF4 is required for class switch recombination and plasma cell differentiation [28], [29]. In T cells, IRF4 is required for lineage commitment by controlling the regulation of several cytokines [30]–[35], but it still remains unknown whether it plays any such role in cytokine expression by B cells. Interleukin-10 (IL-10), despite its initial identification as a T_h_2 specific cytokine, has a broad expression pattern including T_h_1, T_h_2, T_h_17, T_reg_ and B cells. In human B cells, IL-10 has roles in activation, proliferation, immunoglobulin production and differentiation [36]–[39], while the role of murine IL-10 in B cell differentiation is unclear.

In this study, we have identified a key host signaling pathway activated by MHV68 M2 to drive IL-10 expression. We show that M2 signals via the NFAT regulatory network to induce expression of IRF4 which activates the IL-10 promoter. Our study is the first known description of a direct role for IRF4 in IL-10 expression from B cells.

## Results

### Generation of M2 inducible B cell lines that recapitulate M2 induction of IL-10 and IRF4

**Figure 1:**
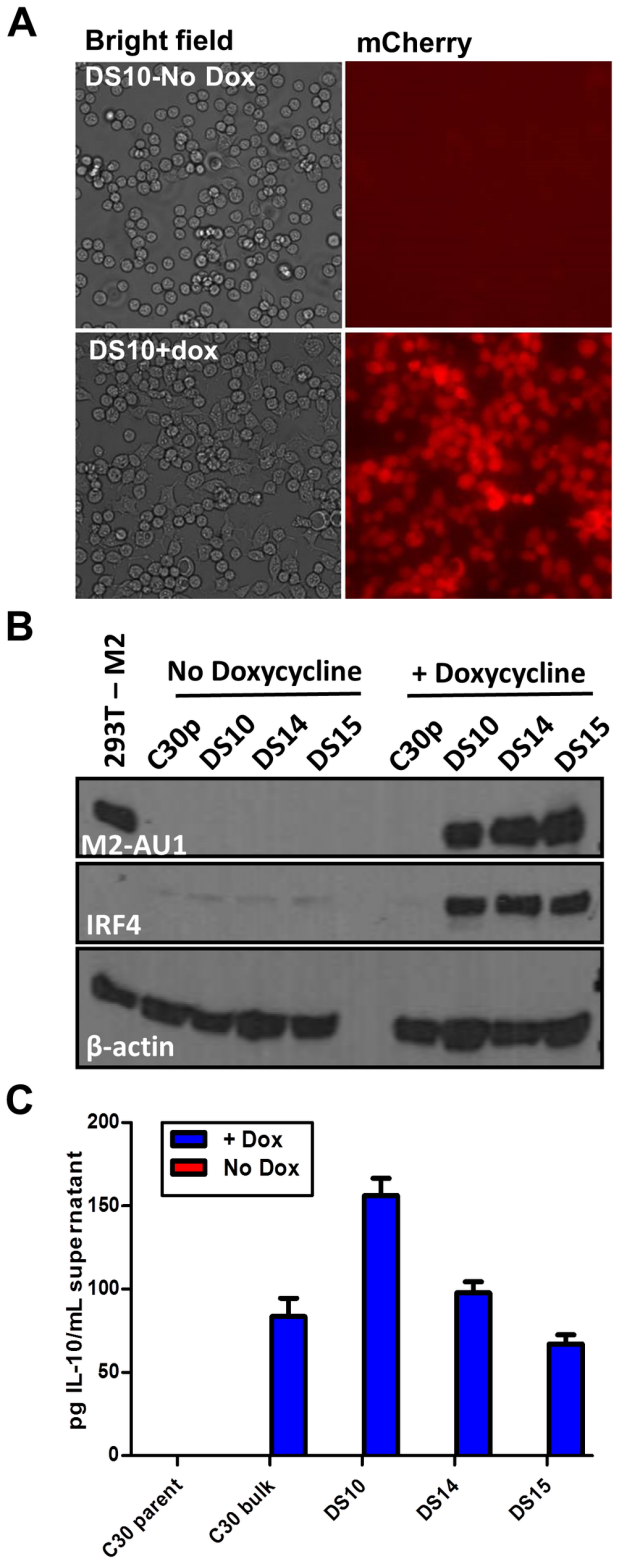
An inducible system to study M2 mediated signaling events. Doxycycline inducible M2 expressing cell lines were generated using the M12 murine B lymphoma cell line. C30p is the parental cell line stably carrying the TetON regulator plasmid, which is used as a negative control. C30bulk refers to the bulk inducible cells prior to limiting dilution analysis. DS10, DS14 and DS15 are individual clones of M2 inducible cell lines. (A) Representative bright-field and mCherry fluorescence images of the same field of DS10 cells that were either left uninduced (top panel) or treated with doxycycline for 48 hours (bottom panel). (B) The indicated cell lines were either left uninduced or induced with doxycycline for 48 hours. Whole cell lysates were harvested and 40 µg protein was resolved by SDS-PAGE followed by immunoblotting for M2, IRF4 and β-actin using antibodies described in Materials and Methods. (C) Supernatants from 2×10^6^ cells that were either doxycycline induced for 24 hours, or left uninduced, were analyzed for IL-10 secretion by ELISA. Data shown is a representative figure from multiple experiments showing similar results.

M2 expression in primary murine B cells leads to high levels of IL-10 secretion in culture supernatants [16]. Since IL-10 production is a common evasion strategy used by human gammaherpesviruses [40], [41], as well as a number of other viruses ([42]–[46] and reviewed in [47]), we wanted to understand and define the roles of key signaling intermediates that are involved in this progression to IL-10 expression. Due to the limited life-span of primary murine B cells in culture and availability of sufficient starting material to perform biochemical studies, we sought to study signaling networks affected by M2 in an established murine B cell line. Notably, our early attempts to overexpress M2 in murine B cell lines were inefficient and confounded by the fact that most murine B cell lines produce significant levels of IL-10. M2 over-expression in these systems resulted in only a modest increase in the level of IL-10, and identification of signaling intermediates was difficult, most likely due to the fact that M2 augments signaling pathways already activated in these cell lines. To overcome these technical challenges, and to express M2 in a temporal and conditional manner, we created inducible cell lines expressing M2 using the Tet-ON inducible system which allows expression of target genes in a doxycycline-dependent manner. For these studies we used the M12 murine lymphoma cell line to create the inducible M2 expression system. The rationale for using this cell line was two-fold: (i) this cell line is a mature B cell line that does not make any endogenous IL-10 and therefore, any IL-10 that we observe would be the result of M2 expression; and (ii) this cell line is amenable to both transient as well as stable transfection methods. To facilitate detection of M2 by immunoblotting, we introduced an AU1 epitope tag at the C-terminus of M2. In addition, the tetracycline regulated expression plasmid employed harbors a mCherry expression cassette upstream of an IRES element that drives expression of the inserted M2 coding sequences. The latter facilitated screening of candidate M2 inducible clones (Figure 1A).

Several candidate clonal cell lines were generated using the inducible expression system, and these were screened for tightly regulated M2 expression. Among several clones tested, clones DS10, DS14 and DS15 were used for further analyses. As a control in all experiments outlined below, we used the parental clonal cell line, C30p, which stably expresses the tetracycline regulator protein (TetON) but does not contain the inducible M2 expression vector. This control was included to assess any effect that addition of doxycycline might have on these cells. Expression of the fluorescent protein mCherry was used as a visual measure for M2 expression, and ensured that all cells in a clonal population responded uniformly to doxycycline (dox) induction (Figure 1A). Furthermore, these cells express M2 only upon doxycycline addition, demonstrating tightly regulated expression of M2 without any detectable leaky basal expression (leaky expression of genes is commonly associated with such inducible systems) (Figure 1B). Importantly, we show that the clones DS10, DS14 and DS15 make IL-10 upon M2 induction by doxycycline (Figure 1C). Since this system recapitulates M2 induction of IL-10 in primary murine B cells, it provided an experimental model to further characterize the signaling events downstream of M2 leading to IL-10 production.

Based on our previous finding describing M2 driven-plasma cell differentiation [15], we also wanted to assess whether expression of M2 in this cell line led to changes in the expression of any plasma cell associated factors, namely IRF4, since we observed a robust increase in both transcript and protein levels in our previously analyses of BCL-1 cells expressing M2 [15]. Consistent with our previous analyses, expression of M2 led to a marked increase in the expression of IRF4 (Figure 1B, middle panel), indicating that M2 driven plasma cell differentiation is potentially a result of M2 modulating the levels of IRF4.

### M2 induction of IL-10 and IRF4 involves activation of the NFAT pathway

**Figure 2:**
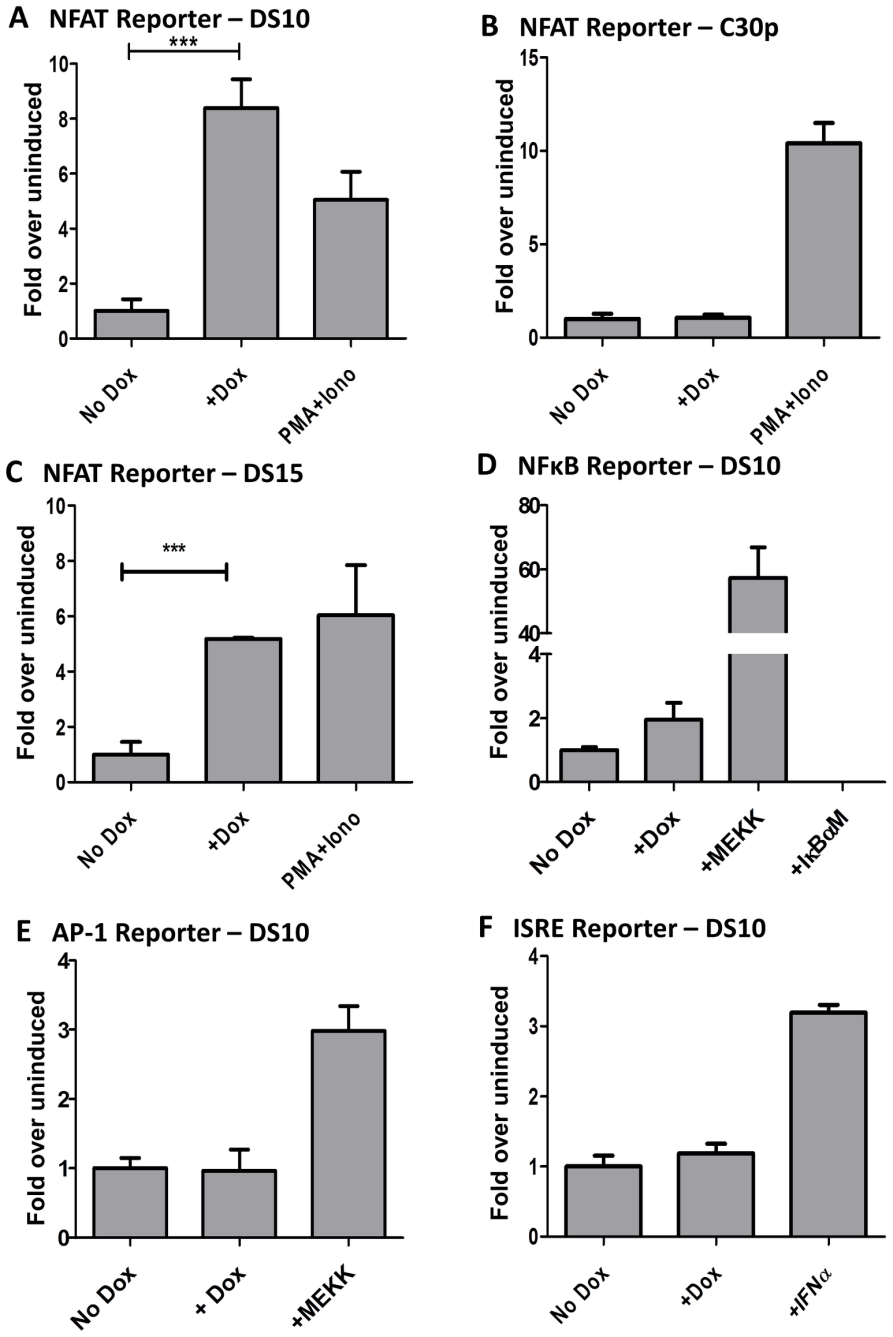
M2 activates the NFAT pathway, but not other major signaling pathways such as NFkB, ISRE, or AP-1. 3×10^6^ cells were nucleofected with 10 µg of the indicated reporter plasmids using the Amaxa solution L or Ingenio (Mirus) electroporation solution as per manufacturer's protocol. After transfection, the cells were divided into two reactions - one half was left untreated and the other half received doxycycline for 48 hours prior to quantitating luciferase activity. Each condition was performed in triplicate wells. Reporter plasmids used are part of the PathDetect cis-reporting system from Agilent technologies. (A–C) Analysis of M2-inducible NFAT activity in DS10, C30p and DS15 cell lines. The pNFAT-luc reporter was nucleofected as described above. PMA (60 ng/mL) and Ionomycin (1 µg/mL) treatment for 6 hours served as a positive control. (D) Analysis of M2 inducible NF-kB activity in the DS10 cell line. pNFκB-luc reporter was nucleofected as described above. pFC-MEKK plasmid and pIκBαM plasmid served as the positive and negative controls respectively. (E) pAP-1 reporter plasmid was used as described above. pFC-MEKK plasmid served as a positive control. (F) pISRE-luc reporter plasmid was used as described above. IFNα treatment for 6 hours served as a positive control. For all analyses, luciferase activity is represented as fold over uninduced ± SEM.

Biochemical characterization of M2 has revealed that M2 acts as an adaptor protein forming a trimolecular complex with Vav and Fyn [18], [48], [49]. Recently, it has been shown that M2 can also bind PLCγ2 - resulting in the inhibition of the Akt pathway [19]. These results, along with *in vivo* work demonstrating that M2 is required for the infected B cells to access the germinal center B cells, strongly suggest that M2 modulates B cell signaling - either by potentiating signals from the BCR or by modulating aspects of BCR-mediated signaling. To gain a better understanding of the major signaling pathways manipulated by M2, we sought to perform a luciferase assay based screen using the PathDetect Signal Transduction cis-reporting system (Agilent technologies). This system employs a collection of inducible reporter plasmids to assess activation of major signal transduction pathways. These reporter plasmids contain a luciferase reporter gene driven by a basic promoter element plus a defined inducible cis-enhancer element. Using these reporters and the inducible cell lines, we analyzed whether M2 induction by doxycycline modulates the key pathways (NFκB, NFAT, AP-1 and ISRE pathways) known to be important in immune response to viruses. When compared to the uninduced cells, the cells that received doxycycline treatment activated the NFAT pathway significantly (Figure 2A). Notably, this activation was of similar or greater magnitude compared to the positive control induction (i.e., treatment with a combination of PMA and ionomycin, well-characterized activators of NFAT pathway), indicating a robust induction of this pathway by M2 (Figure 2A). To verify that this effect was indeed due to M2 expression, rather than an unanticipated side effect of doxycycline addition, we performed the same experiment in the C30p parental cell line. Notably, in the C30p parental cells we failed to observe any impact of doxycycline on the NFAT pathway (Figure 2B), confirming that M2 directly activates the NFAT pathway. We also confirmed M2 induction of the NFAT pathway in another M2 inducible clonal cell line, DS15 (Figure 2C).

Analysis of NFkB reporter revealed weak activation by M2 (Figure 2D). However, it should be noted that established B cell lines in general have high basal levels of NFkB activity. Thus, it is perhaps not surprising that M2 can only modestly enhance this activity. Analyses of the AP-1 and ISRE reporters in the DS10 cell line failed to reveal any impact of M2 on these pathways (Figure 2E and 2F). It is to be noted that others have reported that M2 inhibits the ISRE pathway [50], but we believe that the discrepancy with our data is a reflection of the different cell lines used – perhaps coupled with differences in the experimental methods utilized. Our assay measures the ability of M2 to activate the described pathways in the absence of any external stimuli, while the other study showed that M2 represses ISRE activity in the presence of IFNα [50]. Since we are interested in examining the signaling mechanism(s) utilized by M2 in the absence of external stimuli, this pathway was not explored further. In T cells, several cytokine genes such as IL-2, IL-3, IL-4, TNF-α, GM-CSF, etc. are known to contain NFAT binding sites in their promoter/enhancer regions [51], [52]. To determine whether M2 expression can activate a known NFAT-responsive gene, we performed a reporter assay using an IL-2 promoter driven luciferase construct in DS10 cells. As shown in Figure S1, M2 expression upon doxycycline treatment activates the IL-2 promoter to about 14-fold over uninduced cells. This activity was inhibited upon cyclosporine A (CsA) treatment. Addition of PMA and Ionomycin served as a positive control. Consistent with this data, we have previously shown that transduction of primary murine B cells with a M2 expressing retroviral construct leads to about a 25-fold increase in IL-2 levels in culture supernatants compared to control supernatants on day 4 post-transduction [16]. This suggests that M2 expression in B cells utilizes similar signaling machinery as utilized by T cells to induce cytokine expression and a subsequent immune response.

### M2 activation of the NFAT pathway is dependent on extracellular calcium flux and Src kinase activity

**Figure 3:**
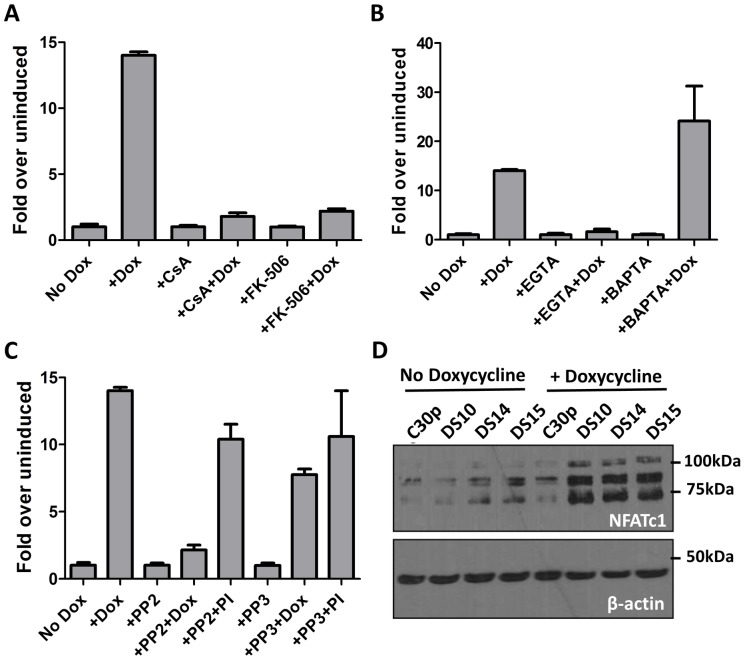
M2 activation of the NFAT pathway is CsA and FK506 sensitive and requires extracellular calcium flux. (A, B and C) Impact of inhibitors on M2-induced NFAT activity. The NFAT reporter assay was performed as in Figure 2A.Doxycycline was added for 48 hours. CsA (500 ng/mL), FK506(1 µM), EGTA(2.5 mM), BAPTA-AM(5 µM), PP2 (10 µM) and PP3 (10 µM) were added 24 hours post-nucleofection and the lysates were assayed for luciferase activity at 48 h post-nucleofection. Data are represented as fold over respective uninduced conditions, setting the uninduced samples in each drug treatment to 1. (D) The indicated cell lines were either left uninduced or induced with doxycycline for 48 hours. Whole cell lysates were harvested and 60 µg protein was resolved by SDS-PAGE followed by immunoblotting for NFATc1 using antibodies mentioned in materials and methods. β-actin was used as a loading control.

CsA and FK506 are commonly used immunosuppressants that inhibit the Ca^2+^ dependent protein phosphatase calcineurin, which in turn inhibits the activation of NFAT pathway resulting in a global inhibition of T cell activation [53]. Therefore, we wanted to study whether the NFAT induction observed upon M2 expression occurred via the Ca^2+^-calcineurin pathway as well. To this end, we performed a NFAT reporter assay in DS10 cells in the presence or absence of CsA and FK506. Doxycycline was added to cells 1 hour after nucleofection followed by addition of CsA or FK506 at 24 hours post-nucleofection. Luciferase activity was measured 48 hours post-nucleofection. To account for any toxicity resulting from the addition of the drugs, data is represented as fold over uninduced for each drug treatment. As shown in Figure 3A, both CsA and FK506 addition inhibited the activation of NFAT pathway mediated by M2, consistent with their roles in inhibition of the NFAT pathway. In contrast to NFκB activation that occurs as a result of intracellular calcium flux immediately following BCR engagement, activation of the NFAT pathway is dependent on a sustained calcium flux mediated by an influx of extracellular calcium (which occurs upon depletion of intracellular calcium stores) [54]. To examine whether M2 induction of the NFAT pathway is mediated by an intracellular or extracellular calcium flux, we performed the NFAT reporter assay in the presence of chelators of intracellular and extracellular calcium. BAPTA/AM [1,2-bis(2-aminophenoxy)ethane-*N,N,N′,N′*-tetraacetic acid tetrakis(acetoxymethyl ester)] is a membrane-permeable ester form of BAPTA that blocks an intracellular calcium flux, whereas EGTA (ethylene glycol-bis(2-aminoethylether)-*N,N,N′,N′*-tetraacetic acid) is a cell impermeable calcium chelator that inhibits an extracellular calcium flux. Addition of EGTA to culture medium abolished the activation of NFAT by M2, whereas addition of BAPTA/AM had no effect (Figure 3B). These results indicate that M2 activation of the NFAT pathway requires a sustained extracellular calcium flux, rather than a transient increase in intracellular calcium.

**Figure 4:**
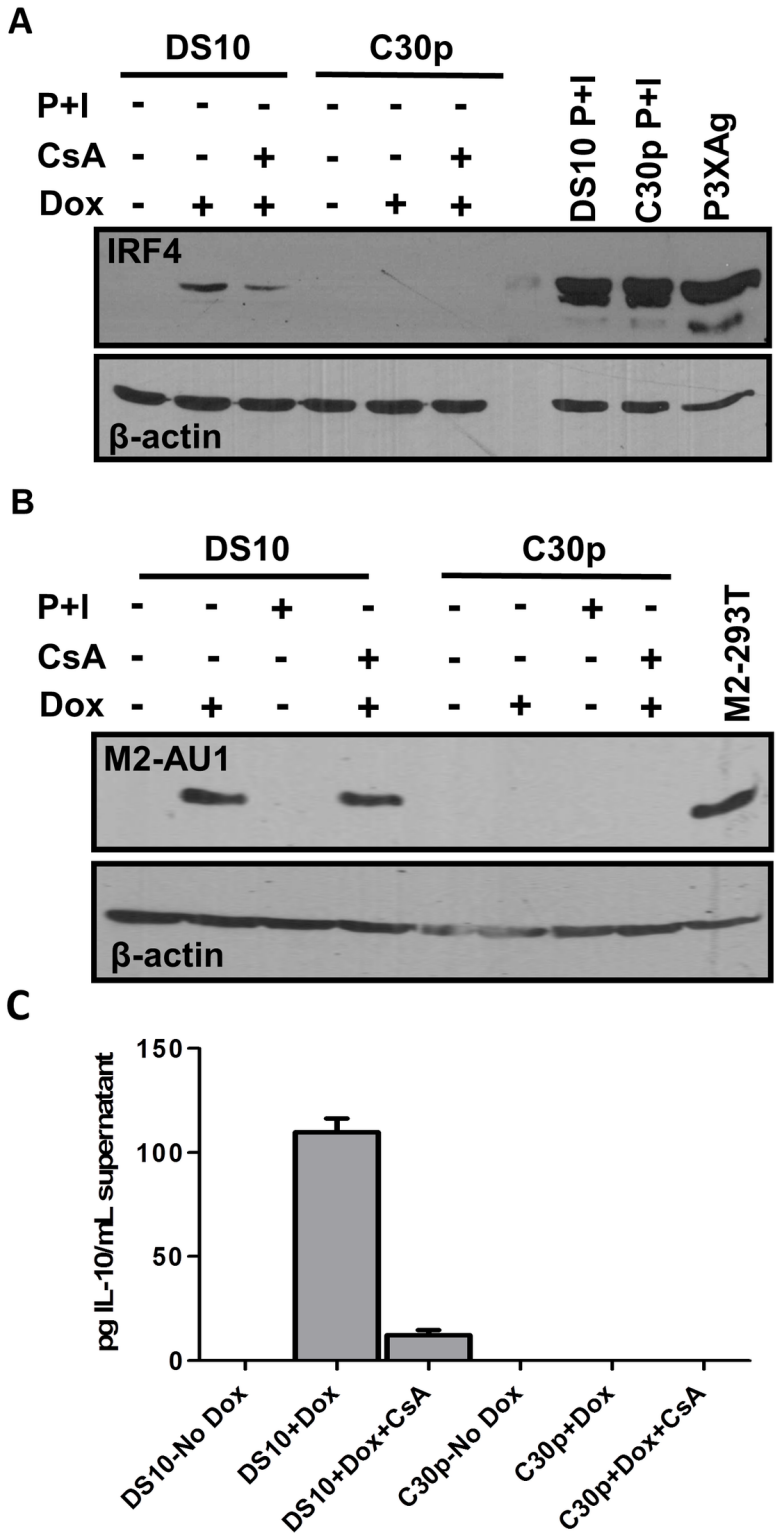
M2 induction of IRF4 by M2 is partially dependent on NFAT pathway. (A) DS10 and C30p cells were either left uninduced or induced with or without CsA for 48 hours in the culture media. Whole cell lysates were harvested at 48 h post induction and 5×10^5^ cell equivalents were analyzed for levels of IRF4 protein by immunoblotting. PMA and Ionomycin treatment for 6 hours served as a positive control for IRF4 induction. P3X63Ag8 cell lysates served as a stimuli independent positive control. (B) Duplicate gel run using whole cell lysates from (A) to verify expression of M2 using antibody for AU1 tag. (C) Supernatants from cells in Figure 4A were analyzed for IL-10 levels by ELISA.

Based on previous studies describing the interaction of M2 with Src family kinase members and PLCγ2 [18], [19], we were interested in determining whether any Src kinase family members play a role in NFAT activity mediated by M2. To this end, we performed a NFAT reporter assay in the presence of PP2, a potent and selective Src kinase family inhibitor. As a negative control, the kinase inhibitor PP3, which inhibits EGFR kinase, was used. Inhibition of Src kinase activity with PP2 abolished M2 mediated NFAT activity (Figure 3C) whereas NFAT activity induced by PMA/ionomycin was unaffected. This result suggests that interaction of M2 with a Src kinase is required for M2-mediated NFAT activation, but is not required for PMA/ionomycin mediated NFAT induction. The addition of PP3 did not affect NFAT activation mediated by M2 or PMA/ionomycin. It is to be noted that none of the drug treatments had a significant effect on the viability of the cells, as measured by trypan blue viability assay (Figure S2). The drug treatments also did not affect the levels of M2 expression as shown in Figure S3A (for PP2 and PP3 treatment), 3C (for EGTA, BAPTA and FK506 treatment) and Figure 4B (for CsA treatment). Furthermore, to test the effect of inhibiting Src-kinase activity on the levels of IRF4 and IL-10, DS10 cells were treated with PP2 and PP3 in the presence or absence of doxycycline. Whole cell lysates were analyzed for IRF4 expression and the supernatants from the cells were tested for IL-10 levels by ELISA. Addition of PP2 resulted in a decrease in IRF4 expression (Figure S3A) correlating with a decrease in IL-10 levels in culture supernatants (Figure S3B). Taken together, these data suggest that interaction of M2 with a Src kinase is one of the initial events that drive induction of NFAT activity, IRF4 expression and IL-10 secretion. Additionally, addition of both EGTA and BAPTA caused a modest decrease in IRF4 levels (Figure S3C, IRF4 short exposure). While it is expected for EGTA treatment to result in decreased IRF4 expression due to a defect in NFAT activation, we were surprised that BAPTA/AM addition also decreased in IRF4 expression. This is potentially due to the fact that addition of BAPTA/AM inhibits activation of the NFκB pathway, another major transcription factor controlling IRF4 expression [55]–[57]. We next assessed whether induction of M2 in the inducible cell lines leads to a change in the endogenous levels of NFAT proteins themselves. Among the different NFAT family members, NFATc1 and NFATc2 are the predominant species expressed in lymphocytes. Therefore, we measured the expression levels of both NFATc1 and NFATc2 in the DS10, DS14 and DS15 cell lines in the presence and absence of doxycycline. As a negative control, the C30p parental cell line was analyzed. We were unable to detect any NFATc2 in these lysates, possibly because this cell line does not express NFATc2 (data not shown). However, we detected low level expression of NFATc1 in the parental C30p and M2-inducible cell lines (Figure 3D, lanes 1–5). M2 expression resulted in an increase in the levels of NFATc1 (Figure 3D, lanes 6–8), indicating that in addition to activating the NFAT pathway, M2 expression also results in an increase in the levels of NFAT protein themselves, potentially driven by an increase in extracellular calcium flux.

### M2 induction of IRF4 expression is partially mediated by NFAT activation

IRF4, a critical player in plasma cell development as well as immunoglobulin class switch recombination [28], [29], is strongly up-regulated upon M2 expression in the inducible cell lines (Figure 1B, middle panel). We hypothesized that M2-driven plasma cell differentiation is associated with IRF4 expression induced via the NFAT pathway. Notably, it has been demonstrated that mice deficient in Cnb1, the regulatory subunit of calcineurin phosphatase, have a defect in expression of IRF4 that is reflected by a decrease in plasma cell response to T dependent antigens [26]. To determine whether the NFAT pathway is involved in the induction of IRF4 expression by M2, we evaluated the expression of IRF4 upon treatment of M2 expressing cells with CsA. Pharmacological inhibition of the NFAT pathway with CsA in DS10 cells led to an ca. 50% decrease in the levels of IRF4 protein induced by M2 (Figure 4A, compare lanes 2 and 3). As a control, PMA/ionomycin treatment induced robust IRF4 expression in both DS10 and the parental C30p cell lines (Figure 4A, lanes 8 and 9). As a stimuli-independent positive control, we used whole cell lysates from a murine myeloma cell line P3X63Ag8 (termed P3XAg) that constitutively expresses IRF4. Consistent with this data, addition of FK506 also caused a decrease in IRF4 levels upon M2 expression (Figure S3C, IRF4 long exposure). Duplicate immunoblots using lysates prepared at the same time and probed for M2 expression (using an antibody to the AU1 epitope tag) confirmed that expression of M2 itself was not affected upon CsA treatment (Figure 4B). In addition, since M2 induces IL-10 in DS10 cells, we analyzed the IL-10 levels in supernatants from cells used in Figure 4A. The addition of CsA to doxycycline treated DS10 cells resulted in a 9-fold reduction in IL-10 levels (Figure 4C). Together, these data suggest that M2 augments/mimics signaling from the BCR, by interacting with and signaling through Src family kinases and/or PLCγ2 to activate the NFAT pathway - leading to a strong up-regulation of IRF4. While the NFAT pathway is not the only pathway required for M2-induced IRF4 expression or IL-10 levels, our data indicates that this pathway plays an important role in M2 function. Importantly, the modest induction of NFkB activity by M2 (Figure 2D) may also contribute, since expression of IRF4 has been shown to be activated by stimuli that activate the NFκB pathway [55]–[57].

### Tyr^120^ and Tyr^129^ are required for M2 mediated NFAT activation and IRF4 expression

**Figure 5:**
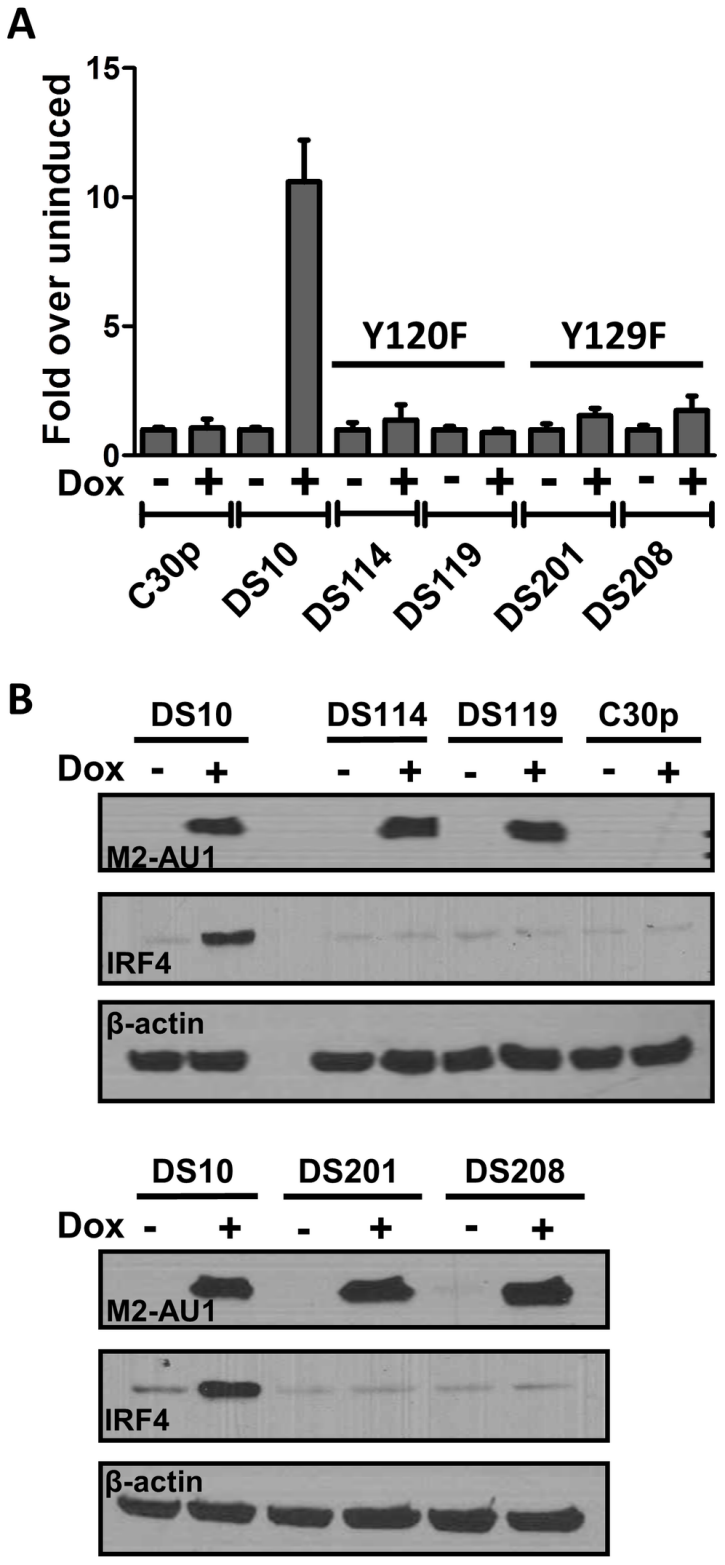
Tyrosines 120 and 129 of M2 are indispensable for M2 mediated NFAT activation and IRF4 expression. (A) 3×10^6^ of the indicated cell types were nucleofected with 10 µg of the NFAT reporter plasmid s using Ingenio electroporation solution as described in Figure 2A. After transfection, the cells were divided into two reactions - one half was left untreated and the other half received doxycycline for 48 hours prior to quantitating luciferase activity. Each condition was performed in triplicate. Luciferase activity is represented as fold over uninduced ± SEM. (B) Whole cell lysates from the indicated cell lines were analyzed for expression of M2, IRF4 and β-actin as described in Figure 1B.

Tyr^120^ and Tyr^129^ of M2 are differentially required for phosphorylation by Src family kinases and for forming multi-protein complexes with SH2 domain containing signaling intermediates such as PLCγ2, SHP2, etc. [18], [19]. Tyr^120^ is the predominant tyrosine that is phosphorylated by Fyn, leading to a trimolecular complex between Vav1, Fyn and M2. While phosphorylation on Tyr^129^ is dispensable for this interaction, it is required for forming complexes with PLCγ2 and SHP2 [19]. In an effort to determine the roles of Tyr^120^ and Tyr^129^ (also called Y120 and Y129), we created M2 inducible cell lines as described above for wild type M2 expression, where the tyrosines 120 and 129 were mutated to phenylalanine (designated Y120F and Y129F). DS114 and DS119 are two independently derived cell lines with the mutation Y120F while DS201 and DS208 are independently derived cell lines with the mutation Y129F. These cell lines were assessed for their ability to activate the NFAT pathway using the NFAT reporter assay. As shown in Figure 5A, cell lines with either Y120F or Y129F mutations failed to activate the NFAT reporter compared to the wild type M2 expressing cell line DS10.Consistent with the hypothesis that NFAT activity is required, at least partly for IRF4 expression, we tested the ability of these cells to express IRF4 upon doxycycline induction of M2 expression. As shown in Figure 5B, none of the mutant cell lines showed an increase in IRF4 expression upon doxycycline induction (Figure 5B, middle panel). It is to be noted that none of the cell lines had a defect in expression of M2 – all expressed M2 at levels equivalent or slightly higher than the DS10 wild type M2 control cell line (Figure 5B, top panel). This suggests that the interaction of M2 with a SH2 domain containing protein(s), such as Src kinases and/or PLCγ2 and/or SHP2, plays a critical role in the initiation of signaling leading to NFAT activation and subsequent IRF4 expression.

### IRF4 expression in B cells leads to IL-10 production

**Figure 6:**
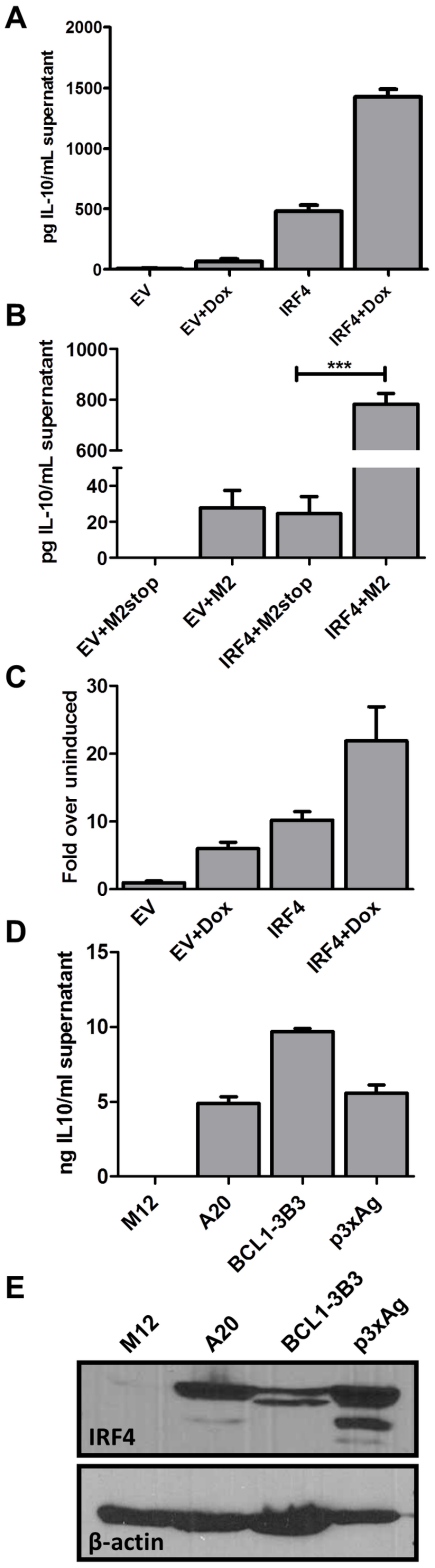
IRF4 expression in B cells leads to IL-10 secretion and M2 synergizes with IRF4 to enhance IL-10 levels. (A) 3×10^6^ DS10 cells were transfected with 10 µg of either an empty vector (pMSCV-IRES-GFP, designated pMIG-EV) or an IRF4 expression vector (pMSCV-IRF4-IRES-GFP, designated pMIG-IRF4) and the reaction was divided into two wells-one well received doxycycline treatment for 48 hours and the other well was left uninduced. Each reaction was performed in triplicates. Supernatants were collected at 48 hours post induction with doxycycline and IL-10 levels were measured by ELISA (B) 1×10^6^ M12 cells per well were nucleofected with an expression vector containing M2 or M2stop, together with either pMIG-EV or pMIG-IRF4.Supernatants were harvested 48 hours after nucleofection and analyzed for IL-10 levels by ELISA. (C) DS10 cells were nucleofected as in (A) above with either pMIG-EV or pMIG-IRF4 along with 2.5 µg of IL-10pCNS9-luc reporter construct. Each reaction was divided into two reactions as above and luciferase activity was measured 48 hours post doxycycline induction. (D and E) The indicated cell types (A20, BCL1-3B3, P3xAg and M12) were plated at a density of 2.5×10^5^ cells in each well of a 6-well plate. Supernatants and whole cell lysates were harvested at 48 hours and analyzed for IL-10 levels by ELISA (D) and IRF4 expression by immunoblotting (E).

IRF4 possesses a multifaceted role in T cell differentiation, particularly in T_h_2, T_h_17 and some aspects of T_reg_ development [30], [34], [52], [58], [59]. Based on this evidence in T cells, and because M2 expression leads to IRF4 as well as IL-10 expression, both of which are CsA sensitive, we wanted to investigate whether IRF4 expression leads to IL-10 production by B cells. To this end, we utilized the M2 inducible DS10 cell line transfected with either an empty vector, pMSCV-IRES-GFP (pMIG-EV), or an IRF4 expression construct, pMIG-IRF4, in the presence or absence of doxycycline. Expression of IRF4 alone resulted in significant levels of IL-10 secretion compared to supernatants from cells transfected with the empty expression vector (EV) (Figure 6A). Furthermore, M2 synergized with IRF4 to enhance the levels of IL-10 present in the culture supernatant. To confirm our findings, as well as to eliminate the possibility that IL-10 expression observed in the DS10 cells was not due to leaky M2 expression in the absence of doxycycline, we overexpressed M2 or M2stop (negative control plasmid) with either the empty expression vector (EV) or an IRF4 expression construct in the M12 B cell line and monitored the levels of IL-10 by ELISA in the culture supernatants at 48 hours post-nucleofection (Figure 6B). Consistent with the results obtained in the DS10 inducible cell line, we observed that either M2 or IRF4 expression alone can induce IL-10, and together they synergize to increase the levels of IL-10 in the culture supernatants (Figure 6B).

In T cells it is known that IRF4 plays a role in activating IL-10 expression [30], [60].Therefore, we investigated whether it holds true in B cells. To assess this, we used a previously described 1.6 Kbp IL-10 promoter construct [61], IL-10pFL, as well as reporter constructs containing the 1.6 Kbp promoter fragment along with upstream enhancer sequences from the IL-10 locus (termed CNS-3 and CNS-9) that have been described to be modulated by IRF4 in T cells [33], [62]. First, we tested the 3 promoter constructs – full length (IL-10pFL), full length plus CNS-3 element (IL-10pCNS-3) and full length plus CNS-9 element (IL-10pCNS-9), for basal and M2-induced activity in the DS10 cells. Among the three constructs, the IL-10pCNS-9 construct exhibited the highest M2-induced activity (Figure S4). Notably, the CNS-9 region contains three putative NFAT binding sites and two putative IRF binding sites [33]. Therefore, we used the IL-10pCNS-9 construct to further assess whether IRF4 modulates the activity of the IL-10pCNS-9 reporter construct in the DS10 cells. Expression of either M2 or IRF4 alone were able to induce activity of this promoter construct, while the combination of IRF4 and M2 further enhanced expression from this reporter (Figure 6C). However, unlike in T_h_2 cells where NFATc2 synergizes with IRF4 in activating CNS-9 enhancer activity [33], co-expression of NFATc1 and IRF4 did not have an effect on IL-10 promoter activity (data not shown).

Since we have previously observed basal IL-10 levels in a variety of murine B cell lines routinely used in our lab, we were interested in determining whether there is a consistent correlation between IL-10 levels and IRF4 expression in established B cell lines. For this analysis we used A20, a mature B cell line known to secrete basal IL-10 [16], BCL-1-3B3 cell line, a leukemic cell line that can be differentiated *in vitro* to plasma cells [63], and P3X63Ag8, a mouse plasmacytoma/myeloma cell line that expresses high levels of endogenous IRF4. We harvested whole cell lysates, as well as culture supernatants, from these cells and performed ELISA for IL-10 and western blot analysis for IRF4 (Figure 6, panels D and E). As expected, the cell lines that expressed IRF4 exhibited high levels of basal IL-10 in their culture supernatants. In contrast, the M12 B cell line does not express any detectable IL-10 or IRF4 (Figure 6, panels D and E). Thus, at least in this limited survey of B cell lines, there is a consistent correlation between IRF4 expression and IL-10 levels. Based on these observations, we conclude that IRF4 can regulate the expression of IL-10 in B cells, a finding that will aid in understanding the roles of IRF4 in plasma cell development, isotype switching and cytokine regulation.

### M2 induction of IL-10 in primary murine B cells involves NFAT activation

**Figure 7:**
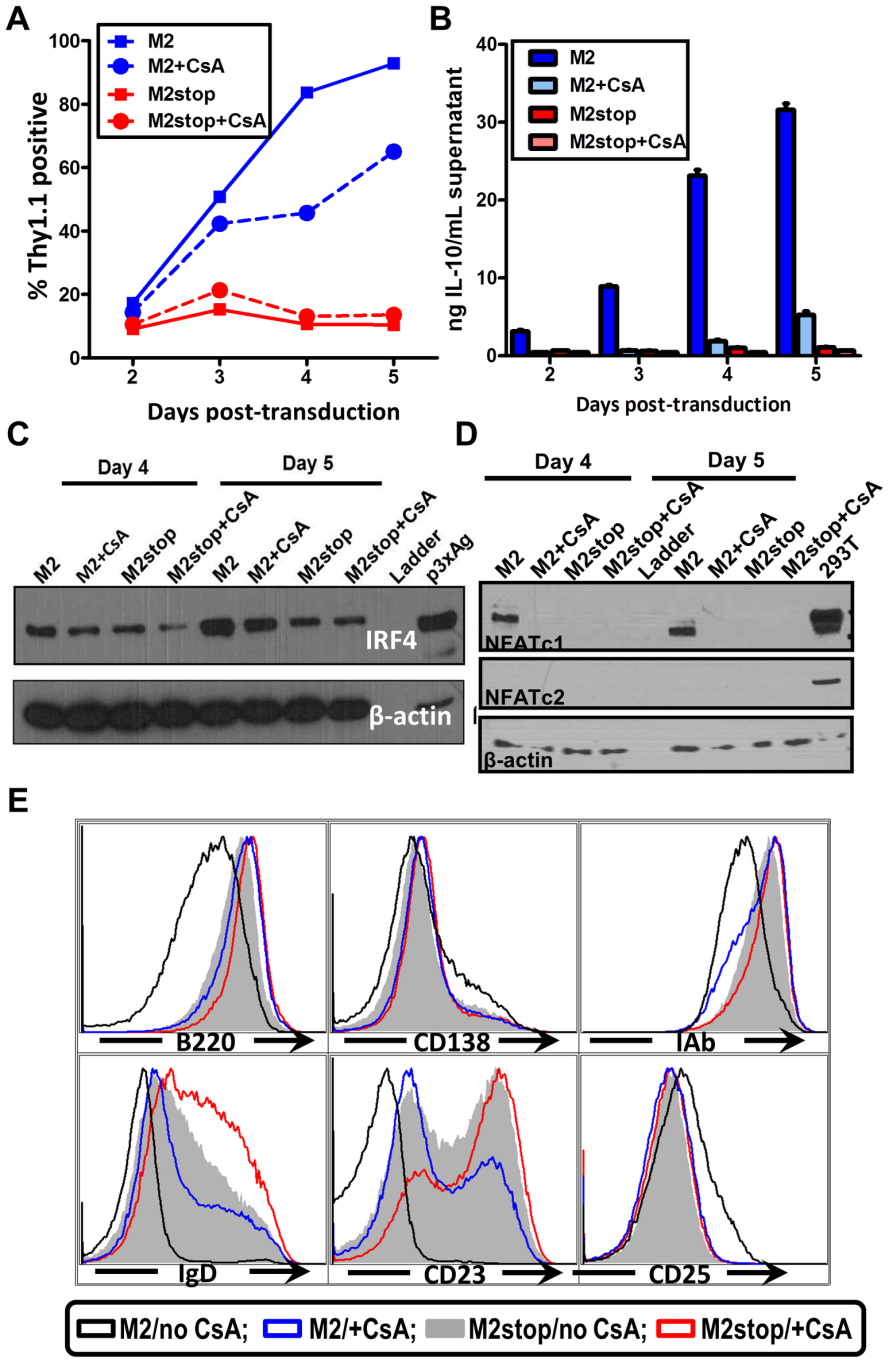
Inhibition of NFAT pathway in primary murine B cells leads to reduced IRF4 expression and IL-10 levels correlating with changes in B cell surface phenotype. (A–E) Primary murine B cells were isolated as indicated in materials and methods, transduced with retroviruses expressing either M2 or M2stop. At the time of transduction, half of the cells received 500 ng/mL CsA and the other half was left untreated. (A) The cells were analyzed for Thy1.1 expression by flow cytometry on days 2 to 5 post-transduction. Cells were first gated on live cells and then gated on Thy1.1 and plotted as frequency of cells that were positive for Thy1.1. (B) Supernatants were collected from the cells used in (A) and analyzed for IL-10 levels by ELISA. (C and D) Whole cell lysates from cells in replicate wells as in (A) were harvested for analyzed for IRF4, NFATc1, NFATc2 and β-actin expression by immunoblotting. 10 µg protein was loaded per well. (E) Gated live cells from the indicated conditions were analyzed for their expression of the surface markers B220, CD138, MHC-IA^b^, IgD, CD23 and CD25. Representative histograms are from day 4 post-transduction depicting one of triplicate samples per condition.

The induction of IL-10 by M2 is most striking in primary murine B cells [16] where M2 expressing cells can induce up to 20-fold more IL-10 than control cells. Therefore, we wanted to confirm that our studies using the inducible cell line can be recapitulated in the primary murine B cells. Since nucleofection of primary murine B cells is extremely inefficient, we cannot perform a NFAT reporter assay in these cells. To get around this limitation, we sought to investigate the effect of pharmacological inhibition of the NFAT pathway in primary murine B cells that express M2 or the negative control. Primary murine B cells were isolated and stimulated overnight with LPS to induce cell proliferation, thereby making them conducive to retroviral transduction. The next day, the cells were transduced with retroviruses expressing either M2 or as a negative control, M2stop. CsA was added to the culture media immediately after transduction and replenished again at day 3 after transduction. Surface Thy1.1 expression was monitored as a surrogate for M2 expression since the retroviral plasmid contains an IRES-Thy1.1 cassette downstream of M2. As we have previously described [16], M2 expressing cultures expanded in culture over time (Figure 7A), reaching almost 90% Thy1.1 positive by 5 days post-transduction compared to M2stop expressing cultures that remained at about 10–20% Thy1.1 positive from days 2 through 5 post-transduction. However, M2 expressing cultures that received CsA started off having similar frequencies of Thy1.1 positive cells as that of untreated cells, but had a striking defect in expansion of Thy1.1 positive population by 4 days post-transduction – ca. 84% of the cells being Thy1.1 positive in cultures that did not receive CsA compared to about 46% of cells being Thy1.1 positive in cultures that received CsA (Figure 7A, compare blue solid and dotted lines). This result indicates that the NFAT pathway is required, at least partially, for the expansion of M2 expressing cells (Figure 7A). In addition, there was a 12-fold decrease in the levels of IL-10 in culture supernatants from cells expressing M2 in the presence of CsA compared to M2 expressing cells without CsA on days 3 and 4 post-transduction (Figure 7B) and a 6-fold decrease on days 2 and 5 post-transduction. M2stop expressing cultures did not have a significant change in frequency of Thy1.1+ cells or IL-10 levels with or without CsA, although the CsA treated cultures expressed slightly lower levels of IL-10 (less than 2-fold), potentially due the requirement of NFAT pathway in LPS mediated B cell proliferation [64]. We also investigated the expression of IRF4 protein in the primary B cells expressing M2 or M2stop in the presence or absence of CsA. Consistent with our observation in the inducible cell line (Figure 4A), primary B cells that were treated with CsA had up to a two-fold decrease in IRF4 expression on days 4 and 5 post-transduction (Figure 6C).

We further examined the levels of NFAT proteins from these primary B cells that received M2 (or M2stop) expressing retroviruses in the presence or absence of CsA. As expected, only M2 expressing cells that did not receive CsA treatment expressed NFATc1, whereas M2stop expressing cells (with or without CsA), and M2 expressing cells that received CsA did not express any detectable levels of NFATc1 (Figure 7D). This is consistent with data from Bhattacharya et al [64], where the authors show that NFATc1 is induced only upon 48 hours of LPS treatment. Since we stimulate the cells with LPS only overnight (12–16 hours) before retroviral transduction, we did not observe any basal NFATc1 levels in M2stop expressing cells. Furthermore, none of the cells expressed any detectable NFATc2 – indicating that this member of the NFAT family of proteins is not expressed in splenic B cells from mice. As a positive control for these immunoblots, 293T cells overexpressing both NFATc1 and NFATc2 were used (Figure 7D, lane 10). This data unequivocally shows that M2 expression alone can directly up-regulate NFAT expression in primary murine B cells, implicating a direct role for NFAT in M2-mediated functions.

M2 expression in primary B cells leads to changes in surface phenotype that closely mirror that of an activated, pre-plasma memory B cell phenotype (CD19^+^, B220^low^, CD138 ^low^, sIgD^−^) [16]. Therefore, we analyzed whether CsA treatment had any effect on M2-mediated differentiation of B cells. Predictably, addition of CsA perturbed the differentiation of M2 expressing cells into a pre-plasma cell type. As shown in Figure 7E, M2 expressing cultures that received CsA were similar to M2stop expressing cells with respect to their expression of activation markers and plasma cell differentiation markers. Namely, MHC-IA^b^ down-regulation and up-regulation of CD25 were inhibited by CsA addition (compare black vs blue histograms in Figure 7E). B220 levels in M2 expressing cells that received CsA were similar to that of M2stop expressing cells, whereas M2 expressing cells without any CsA had strikingly down-regulated B220 levels. As noted in our previous work [16], CD138 levels were only modestly up-regulated, indicating that the cells have not fully differentiated into a plasma cell phenotype. However, M2 expressing cells without CsA addition were trending towards up-regulating CD138 expression compared to M2 expressing cells that received CsA or M2stop expressing cells (with and without CsA) (Figure 7E). In contrast, sIgD and CD23 expression were strongly affected by addition of CsA to both the M2 and M2stop expressing cultures. M2 expressing cells that did not receive CsA treatment had completely down-regulated both sIgD and CD23, whereas CsA addition resulted in a considerable number of cells that still expressed sIgD and CD23 (compare black open histograms vs blue open histograms). While M2stop cultures were already severely affected in their ability to down-regulate both sIgD and CD23, CsA addition further affected this phenotype (compare grey shaded histograms vs red open histograms). This is partly due to the fact that LPS drives B cells to a plasmablast phenotype and addition of CsA in these cultures affects LPS driven B cell activation and differentiation as well. Consistent with our report, in humans, CD23 levels are down-regulated in plasma cells in an IRF4 dependent fashion [57], [65]. Taken together, these data convincingly support a model where M2 activates the NFAT pathway to increase NFATc1 levels leading to increased expression of IRF4, resulting in differentiation of M2 expressing cells into a pre-plasmablast phenotype accompanied by a significant increase in IL-10 secretion.

### IRF4 plays a critical role in both the establishment of MHV68 latency and virus reactivation

**Figure 8:**
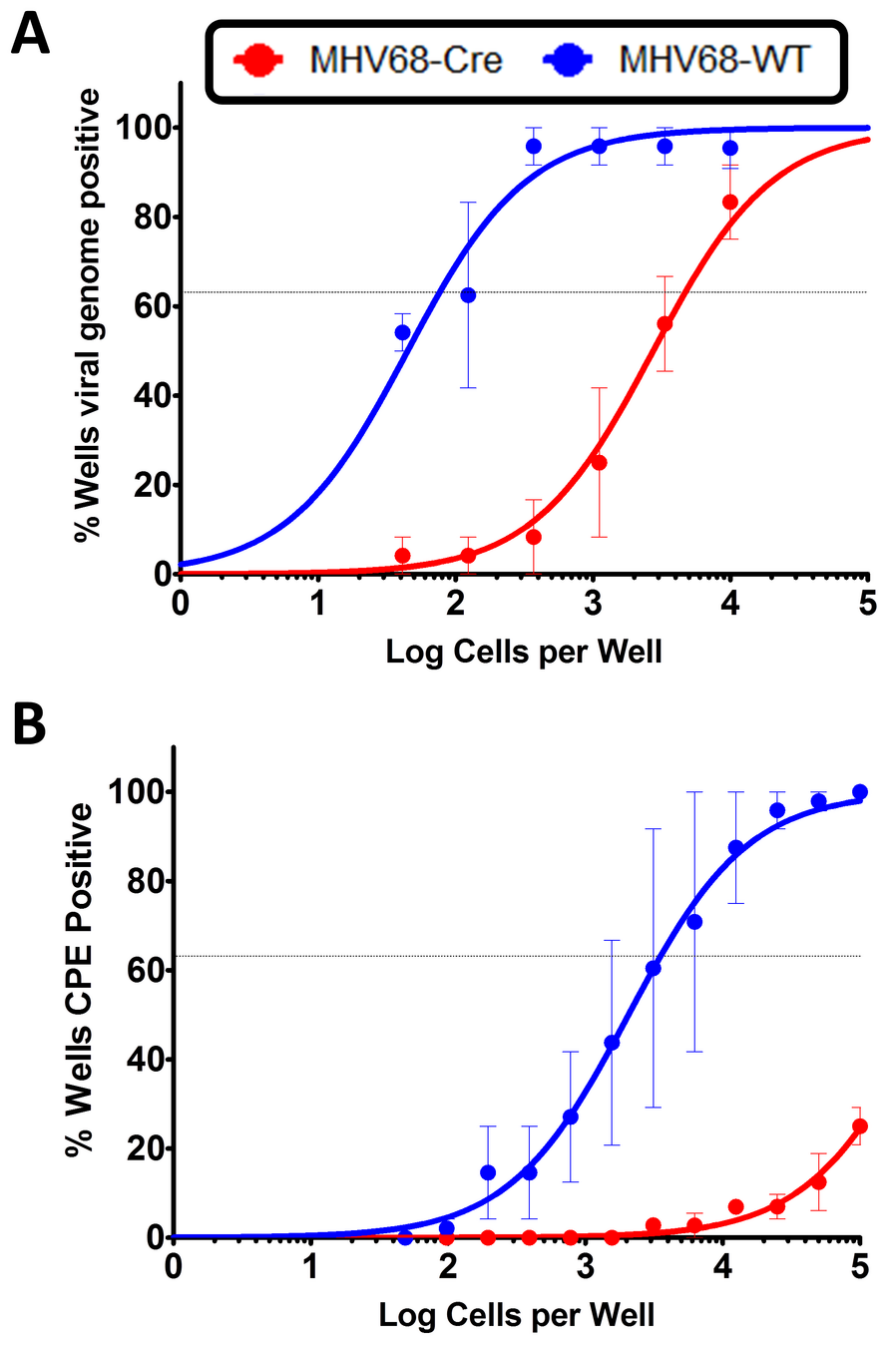
IRF4 is required for establishment of MHV68 latency as well as for reactivation from latency. IRF4^fl/fl^ mice were infected with 1000 PFU of either MHV68-Cre or MHV68-WT virus intranasally. On day 16 post infection, splenocytes were analyzed by limiting dilution analyses to determine (A) the frequency of virus capable of establishing latency and (B) the frequency of virus capable of reactivating from latency.

To gain an understanding of the role of IRF4 in MHV68 infection within the context of the infected host, we utilized IRF4 conditional knockout mice (IRF4^fl/fl^ mice) in which exons 1 and 2 of the IRF4 locus are flanked by loxP sites [29]. Expression of Cre-recombinase in these mice results in the deletion of sequences flanking the loxP sites, thereby leading to deletion of IRF4 specifically in Cre-recombinase expressing cells. We have previously developed and characterized a recombinant MHV68 virus that expresses a Cre-recombinase cassette in a neutral locus of the MHV68 genome, between ORF27 and ORF29b [8]. Infection of C57BL/6 mice with this virus behaves similarly to infection with MHV68-WT virus with respect to acute replication and establishment of latency [8]. In order to study the role of IRF4 in MHV68 infected cells, we infected IRF4^fl/fl^ mice intranasally with 1000 PFU of either MHV68-Cre or MHV68-WT (wild type) virus. On day 16 post-infection, splenocytes were analyzed for establishment of latency and reactivation from latency by limiting dilution PCR and limiting dilution ex-vivo reactivation analyses respectively. As shown in Figure 8A, deletion of IRF4 in MHV68-Cre infected cells leads to a 60-fold decrease in establishment of latency in the spleen (1 in 4710 splenocytes contain latent viral genomes) compared to infection with a MHV68-WT virus (1 in 77 splenocytes harbor latent genomes), indicating a critical role for IRF4 for establishment of MHV68 latency in the spleen. Consistent with this observation, *Ochiai et al* [66] have demonstrated that mice with conditional deletion of IRF4 in CD19 expressing B cells, fail to develop germinal centers. Furthermore, they showed that this defect was intrinsic to IRF4−/− B cells by performing mixed bone marrow chimera studies with IRF4+/+ and IRF4−/− progenitors. Thus, they concluded that IRF4 plays a vital and essential role in germinal center formation and differentiation. Therefore, it is not surprising that deletion of IRF4 in MHV68 infected cells leads to a latency defect since germinal center B cells are the major latency reservoir for MHV68 infected cells. Strikingly, the efficiency of virus reactivation from latency was severely compromised in mice infected with MHV68-Cre virus (1 in 526,017 splenocytes capable of reactivating virus) compared to mice infected with MHV68-WT virus (1 in 3540 splenocytes capable of reactivating virus) (Figure 8B). This 150-fold reduction in viral reactivation capacity in cells infected with the MHV68-Cre virus is reminiscent of a defect in reactivation that we consistently observe in mice infected with the M2 null virus [13], [17]. Thus in the absence of IRF4 from infected cells, the kinetics of viral latency and reactivation resemble that of a M2 null virus, persuasively arguing for a critical role for IRF4 in M2 mediated functions *in vivo* as well.

**Figure 9:**
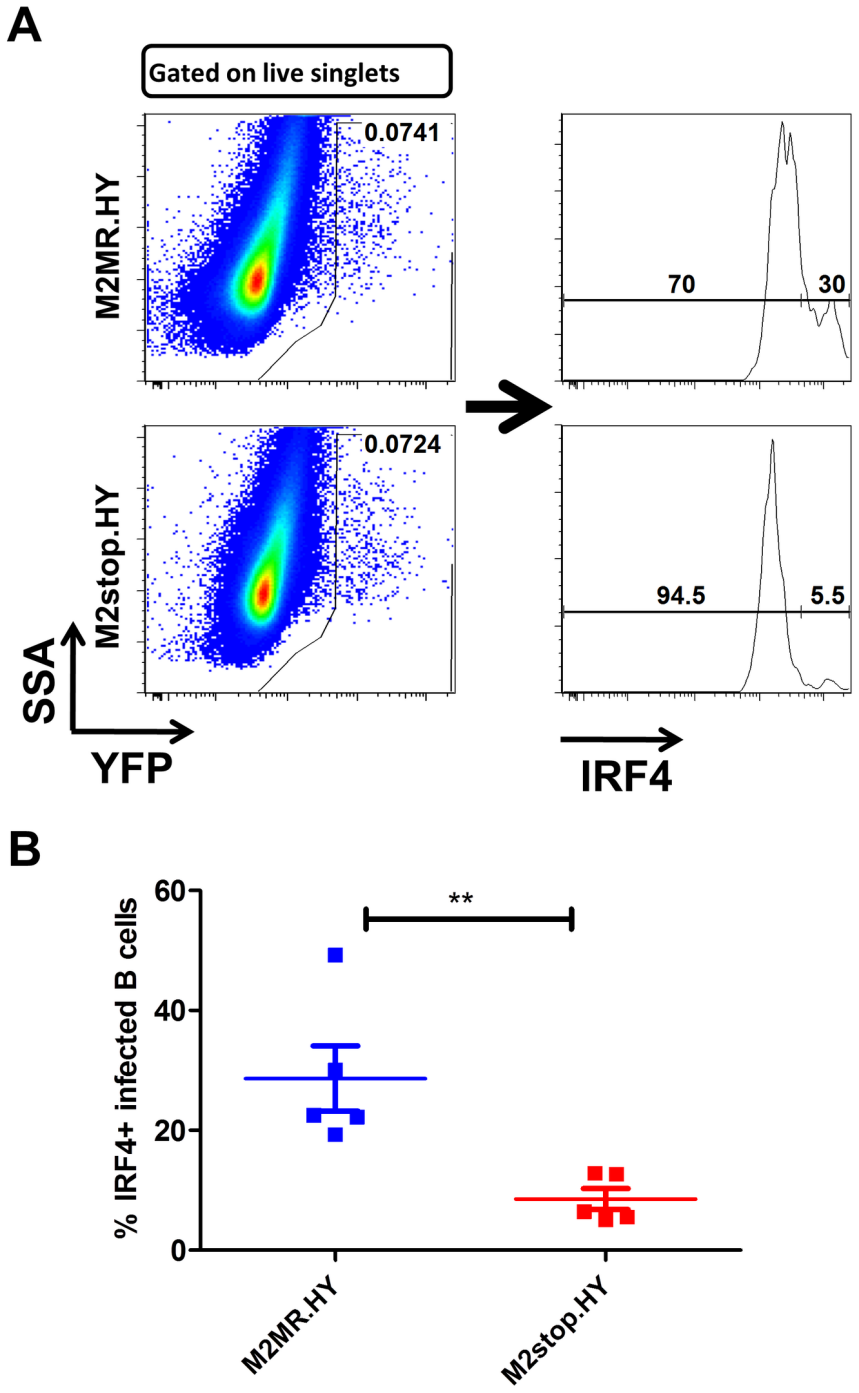
M2stop infected mice exhibit a decreased frequency of infected B cells expressing IRF4. C57BL6/J mice were infected intraperitoneally with 1000 PFU of the indicated viruses and splenocytes were analyzed on day 14 post infection. (A) Representative flow plots illustrating the gating strategy. Splenocytes were gated to remove doublets following gating on the live cell population using a live/dead stain (Materials and Methods). (B) Frequency of live, singlet YFP+ cells that were IRF4 positive between M2stop.HY and M2MR.HY viruses. Data shown is from individual mice with five mice per virus group.

We have previously shown that in C57BL6/J mice infected with a M2 null virus there is a significant decrease in the percentage of infected cells exhibiting a plasma cell phenotype (B220^low^CD138^hi^) [15]. To extend our observation that M2 induces IRF4 expression, which is required for plasma cell differentiation, we have taken advantage of the development of a recombinant MHV68 that allows tracking of infected cells *in vivo*. This approach utilizes a transgenic MHV68 virus that expresses an eYFP-H2B fusion protein (MHV68-H2bYFP) [67]. Using the λRed mediated galK recombineering [9], [68], we generated a new M2 null virus on the MHV68-H2bYFP backbone (M2stop.HY). As a control, we also created a genetically repaired marker rescue virus (M2MR.HY). We have extensively characterized the M2MR.HY and M2stop.HY viruses and have confirmed that they behave like the M2WT-MHV68 and M2stop-MHV68 viruses, respectively (data not shown) [13], [15], [16]. To address whether there is a defect in IRF4 expression *in vivo* in MHV68 infected B cells in the absence of M2 expression, we infected C57BL6/J mice with 1000 PFU of either M2stop.HY or M2MR.HY viruses. On day 14 post-infection, splenocytes were harvested and analyzed for IRF4 levels by intracellular staining coupled with flow cytometry. As a control for YFP expression, we used WT-MHV68 (without an eYFP-H2b expression cassette) virus infected splenocytes. We also used WT-MHV68 infected cells to stain with an isotype control for IRF4 intracellular staining. We analyzed splenocytes from individual mice infected with either the M2stop.HY or M2MR.HY viruses for expression of IRF4 in infected cells (live cells/YFP+/IRF4+). Notably, M2stop.HY infected animals have a significant defect in the frequency of infected cells that express IRF4 compared to M2MR.HY infected animals; only ca. 8% of YFP+ cells in M2stop.HY infected animals express IRF4 compared to ca. 28% in M2MR.HY infected animals (Figure 9). Consistent with our previous finding that M2stop infected mice have a defect in virus infection in plasma cells, we now show that this defect is likely due to a significant defect in the frequency of virus infected B cells expressing IRF4.

## Discussion

Plasma cell differentiation-driven virus reactivation is a common theme in gammaherpesviruses biology [15], [69]–[71]. In contrast to MHV68 where we have previously shown that the viral M2 antigen can promote plasma cell differentiation [15], for both EBV and KSHV there is no information regarding how plasma cell differentiation of latently infected B cells is regulated. Although there are no obvious homologs of the MHV68 M2 gene in the human gammaherpesviruses, it seems reasonable to speculate that EBV and KSHV encode viral gene products that are capable of promoting plasma differentiation – ensuring that virus reactivation occurs at both the appropriate time and anatomical site(s) (e.g., mucosal surfaces). Here, we describe the signaling pathway that ensues upon expression of M2. Expression of M2 in primary murine B cells results in secretion of IL-10, a cytokine associated with plasma cell differentiation in humans [36] and in mice [72]. We therefore hypothesized that identifying the mechanism underlying M2-driven IL-10 production will provide clues about pathways required for plasma cell differentiation driven virus reactivation. As a first step, we created an inducible cell line expressing M2 in a tightly regulated temporal manner. As described in (Figure 1C), this cell line makes IL-10 only when M2 expression is induced by doxycycline treatment, thereby providing us a means to study M2 mediated IL-10 production in a systematic fashion.

M2 activates the NFAT pathway (Figure 2, panels A & C), but does not appear to activate the NFκB, AP-1 or the ISRE pathways (Figure 2D–2F). The NFAT pathway, along with NFκB pathway, represents one of the primary pathways utilized by lymphocytes to transduce several signals required for proliferation, survival, maintenance and differentiation. Although it is surprising that M2 did not significantly activate the NFκB pathway (Figure 2D), this is potentially due to the fact that this cell line has high basal NFkB activity - likely necessary for survival and growth of this cell line. Notably, we have carried out a time-course analysis following induction of M2 expression to confirm that the absence of NFkB activation is not a time-sensitive phenotype (data not shown). The inability of M2 to activate the AP-1 and ISRE pathways (Figure 2, panels E & F) suggests that M2 does not induce a generalized signaling cascade as in the case of BCR activation, but rather selectively activates the NFAT pathway to facilitate its signals.

M2 contains two tyrosines, Tyr^120^ and Tyr^129^ that are differentially required for the formation of a trimolecular complex consisting of Vav, Fyn and M2 [18]. It also contains nine PxxP motifs, some of which are capable of binding SH3 containing proteins [17], [19], and shown to be important *in vivo*. Interestingly, a mutant virus with both Tyr^120^ and Tyr^129^ mutated to phenylalanine has a defect in reactivation from latency, fitting our hypothesis that the interaction of the tyrosines with Src family kinases is crucial for the signals driven by M2. Consistently, M2 inducible cell lines with mutations of either tyrosine 120 or 129 to phenylalanine fail to activate the NFAT pathway and fail to up-regulate IRF4 expression (Figure 5). Therefore, M2 seems to function predominantly as a scaffolding protein that forms multiprotein complexes mimicking or perpetuating signals from the BCR. Consistent with this hypothesis, published studies suggest that M2 forms a microsignalosome complex similar to that observed upon BCR crosslinking [19].

Interestingly, recent studies have shown mice lacking Stromal Interaction Molecules 1 and 2 (STIM1 and STIM2 - the ER Ca^2+^ sensor required for sensing depletion of ER Ca^2+^ stores) are defective in BCR induced SOC (Store Operated Calcium) influx, proliferation and NFAT activation. Also, these mice have a striking defect in IL-10 production, indicating that extracellular calcium flux mediated IL-10 production requires NFAT activity [73]. Their finding is the first report linking B cell derived IL-10 to NFAT activation, although they characterized these IL-10 producing B cells as B_regs_ – a subset of B cells that play an important role in controlling autoimmune responses. While, several other reports have identified a regulatory role for B cell derived IL-10 in mice [72], [74]–[79], we have not characterized whether M2 expressing B cells are phenotypically similar to B_reg_ cells described in these studies and whether these cells possess any regulatory capacity, namely limiting autoimmunity, implicated in these studies.

This study is the first description of a specific role for IRF4 in B cell derived IL-10. *Maseda et al* [72] have shown using IL-10 reporter mice that B_reg_ cells that transcribe IL-10 have increased transcripts of the plasma cell associated factors Blimp-1 and IRF4. However, there was no evidence suggesting that IRF4 was responsible for driving the B_reg_ cells to transcribe IL-10. Here, we show that IRF4 expression is sufficient to induce IL-10 in culture supernatants of DS10 and M12 cell lines, which do not normally express IRF4 or IL-10 (Figure 6, panels A & B). Concomitant with this finding, IRF4 activates an IL-10 promoter construct containing an enhancer element (Figure 6C). Strikingly, M2 synergizes with IRF4 to activate the promoter, as well as, IL-10 secretion (Figure 6A–C). Interestingly, since IRF4 by itself is a weak DNA binding protein, IRF4 synergizes with NFAT family members in activating the promoters of IL-4 and IL-10 in T cells [33], [52]. Therefore, we expected IRF4 to synergize with NFAT family members to activate the IL-10 promoter in B cells, but failed to observe any such synergy with NFATc1 (data not shown). Nevertheless, since M2 synergizes with IRF4 to activate the IL-10 promoter and because M2 expression results in an increase in NFATc1 levels (Figure 3D), it is likely that the synergy with M2 is indeed mediated by NFATc1. While it is formally possible that M2 could possess a transcription factor-like function, it seems unlikely since there is no evidence so far suggesting a direct role for M2 in transcriptional regulation. Supporting this idea is the fact that M2 does not localize to the nucleus in B cells [80], although it does so in other cell types.

IRF4 is a lymphocyte-specific member of the interferon regulatory factor (IRF) family of proteins, well known for its role in plasma cell development and isotype switching in B cells, as well as transcriptional regulation of B cell molecules, namely Igκ light chain enhancer, CD23,CD20 and MHC-I (reviewed in [27] and [81]). IRF4 is also involved in the regulation of several T-cell derived cytokines, especially those involved in T_h_2 development and in T_h_17 differentiation [31], [35], [58], [59]. Along with Blimp-1, it is required for the differentiation and effector function of T_reg_ cells, displaying a multifactorial role in T cell differentiation and regulation [30], [35], [56], [58], [63], [82]. Moreover, the role of IRF4 in development of T_h_2 lineage is in part due to its role in regulation of the IL-10 promoter and IL-4 promoter [30], [32], [33], [52]. Therefore, while IRF4 is extensively studied in T cell lineage commitment owing to its ability to regulate transcription of several cytokines, our report is the first characterization of IRF4 in modulation of B cell specific cytokines. This opens the possibility to explore whether B cell derived cytokines utilize similar mechanisms of regulation as observed in T cells.

Since LPS treatment drives plasmablast differentiation by up-regulating IRF4, Blimp-1, XBP-1, CD138 (syndecan-1) and immunoglobulin secretion, it is difficult to completely abolish IRF4 levels in our experiments using retrovirally transduced primary B cells [e.g., analyses of M2 expression in the presence or absence of CsA (Figure 7E)]. Retroviral transduction requires cell division and hence we transiently expose primary murine B cells to LPS overnight, prior to transduction with the MSCV vectors encoding M2 or M2stop. Therefore, CsA treatment potentially only reduces the levels of IRF4 that is newly synthesized following M2 expression versus that made in response to LPS stimulation. Consistently, this is reflected in IL-10 levels - in cultures that express M2 in the presence of CsA, the IL-10 levels are not decreased to basal levels as seen with M2stop expressing cultures (with or without CsA) (compare light blue bars to red bars in Figure 7B), indicating that a significant portion of IL-10 being made on days 4 and 5 post-transduction is heavily dependent on sufficient IRF4 expression by these cells. In this aspect, the data with M2 inducible B cell lines is robust since CsA is added at the time of addition of doxycycline and hence any IRF4 that is made is potentially through a NFAT-independent pathway (Figure 4A) (likely via the NFκB pathway). Of note, the LMP1 oncogene of EBV induces IRF4 expression in human B cells and is required for the proliferation of these cells [83]. While the authors did not look at IL-10 levels, several independent reports describe the association of LMP1 expression with increased IL-10 levels [84]–[88]. Together, these data indicate that LMP1, somewhat similarly to M2, can induce IRF4 as well as IL-10.

Of particular interest is the *in vivo* data obtained using IRF4^fl/fl^mice (Figure 8). Since the system we utilized knocks out IRF4 only in infected cells, it is to be noted that IRF4 expression is intact in other cells that are uninfected which may be sufficient to elicit a modest germinal center response. However, this defect in latency does not explain the striking defect observed in reactivation from latency upon infection with a MHV68-Cre virus, thereby attributing a specific reactivation defect in addition to the latency defect. This piece of data further tightly links plasma cell differentiation to reactivation as observed with the M2 null virus [15]. Based on observations from *Ochiai et al* [66], it would be interesting to study the course of virus infection in mice with IRF4 deleted in B cells specifically by crossing the IRF4^fl/fl^ with CD19-Cre mice which express the Cre-recombinase from the CD19 promoter, thereby eliminating IRF4 specifically in B cells. Since these mice have a defect in germinal center response, we would speculate that infection of these mice with MHV68 will cause a significant defect in establishment of latency, as well as reactivation from latency.

Interestingly, the NFAT pathway has been implicated, both directly and indirectly, in EBV and KSHV infection. Indeed, induction of a calcium flux by PMA and ionomycin is a standard treatment for initiation of the viral lytic cycle. In addition, induction of the lytic cycle of EBV by surface immunoglobulin crosslinking can be blocked by CsA and FK506 [89], which acts to inhibit Ca^2+^ dependent transcription of the lytic switch gene BZLF-1 (Zta). This CsA sensitivity was mediated by calcineurin and NFATc2 in synergy with TPA and/or the EBV induced Ca^2+/^calmodulin dependent kinase type IV/Gr [90]. In KSHV, ionomycin is a known inducer of Rta (the conserved immediate-early gene sufficient to drive reactivation) – it has been published that in the PEL cell line, BCBL-1, and DMVEC (immortalized human dermal microvascular endothelial cells) calcium mobilization can reactivate latent KSHV [91]. Furthermore, KSHV encodes K1, an oncogenic protein capable of transforming rodent fibroblasts, that contains a variant ITAM motif at its C-terminal cytoplasmic tail that can initiate calcium flux in B cells and subsequent increase in cellular tyrosine phosphorylation leading to activation of NFAT and AP-1 [1], [11], [12], [92]. Additionally, NFAT and AP-1 activation was associated with an increase in production of IL-10 and monocyte-derived cytokine (MDC) in BJAB cells [11]. However, unlike M2, K1 overexpression is insufficient to drive KSHV reactivation from PEL cells [93]. Taken together, despite discernible homology to any known cellular or viral protein, MHV68 M2 shares functional similarities with signaling molecules encoded by EBV and KSHV.

**Figure 10:**
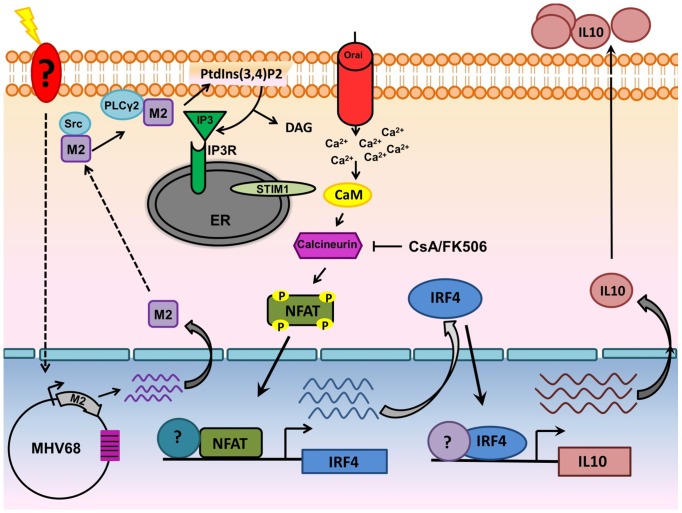
Model of M2 mediated IL-10. Upon BCR engagement, multiple protein kinases including Src family kinases (Lyn,Fyn,Src,Blk,Yes), Syk, Btk get activated leading to the activation of PLCγ2.Activated PLCγ2 catalyzes the hydrolysis of phosphatidylinositol-4,5-biphosphate [(PtdIns(4,5)P2] to DAG and IP3. Binding of IP3 to its receptor on the ER membrane leads to release of ER Ca^2+^ stores. However, this increase in Ca^2+^ is only transient and following depletion of ER Ca^2+^ stores, an influx of extracellular Ca^2+^ occurs (termed as SOCE-Store Operated Calcium Entry). This prolonged and sustained increase in Ca^2+^ leads to activation of the Calcineurin-NFAT pathway. In the case of M2, *Miranda et al* have shown that M2 can interact with Fyn and PLCγ2. Our model suggests that interaction of M2 with proximal membrane signaling molecules such as Src kinase and/or PLCγ2 potentially mimics events that occur upon BCR engagement resulting in the Ca^2+^ mediated activation of the NFAT pathway. Induction of IRF4 by M2 is dependent, at least partially by the activation of NFAT pathway. IRF4 can activate regulatory elements in the IL-10 promoter locus (termed CNS-Conserved Noncoding Sequences) in T cells and in our case, in B cells as well. These series of events leads to eventual increase in the levels of IL-10 observed upon induction of M2.

In conclusion, we propose a model in which interaction of M2 with Vav, Src and/or PLCγ2 initiates a cascade of events that result in activation of the NFAT pathway. This in turn leads to increased levels of IRF4 protein, which activates IL-10 promoter elements resulting in increased IL-10 expression (Figure 10). Notably, M2 induction of IRF4 expression likely involves the activation of another pathway(s) in addition to the NFAT pathway, since CsA treatment does not completely block M2 induction of IRF4 expression (Figure 4A and 7C) or IL-10 production (Figure 4C and 7B). Of specific interest is the role of NFAT and IRF4 in gammaherpesvirus biology. Since immunocompromised patients receiving CsA and FK506 are at high risk for lymphoproliferative disorders associated with EBV and KSHV, further insights into these pathways may eventually aid in the development of novel strategies for better controlling virus reactivation and expansion of latently infected cells in such settings.

## Materials and Methods

### Ethics statement

This study was carried out in strict accordance with the recommendations in the Guide for the Care and Use of Laboratory Animals of the National Institutes of Health. The protocol was approved by the Emory University Institutional Animal Care and Use Committee, and in accordance with established guidelines and policies at Emory University School of Medicine (Protocol Number: YER-2002245-031416GN).

### Cell lines

The M12 murine B cell lymphoma cell line was used as the parent cell line for the development of an inducible cell system that expresses M2 conditionally. The inducible cell line was made according to manufacturer's instructions (Clontech). Briefly, the M12 cell line was transiently transfected with the pCMV-TetON regulator plasmid containing the TetON regulator driven by a CMV IE promoter. The cells were selected with G418 at a concentration of 1 mg/mL. Stable cells that were resistant to G418 were expanded and plated in a limiting dilution manner to obtain single cell clones. Clonal cells were screened for their ability to express TetON by a transient transfection with the pTRE3G-luciferase plasmid that contains the tetracycline response element driving luciferase activity. Clones that exhibit highest luciferase activity only in the presence of doxycycline (a tetracycline analog) were chosen. This cell line designated C30p, was used as a parental cell line to account for any phenotypes associated with doxycycline treatment. C30p cells were transfected with the pTRE3G-mCherry-IRES-M2 plasmid along with a linear hygromycin selection marker. Stable bulk cells (termed C30bulk) that were resistant to hygromycin (400 µg/mL) were plated in a limiting dilution manner to obtain single cell clones. Clonal cells were screened by doxycycline (500 ng) treatment followed by visualization of mCherry expression. Cell lines A20, M12 and its derivatives (C30p, DS10, DS14 and DS15) were grown in complete RPMI-1640 (cRPMI) supplemented with 10% FCS, 100 U/mL penicillin, 100 mg/mL streptomycin, 2 mM L-glutamine and 50 mM β-mercaptoethanol. P3X63Ag8 (ATCC-TIB-9), BCL1-3B3 and primary murine B cells were cultured in complete RPMI-1640 along with 10 mM non-essential amino acids, 1 mM sodium pyruvate and 10 mM HEPES. NIH 3T12 cells used to grow virus stocks and Mouse Embryonic Fibroblasts (MEFs) used to measure reactivating virus from splenocytes were maintained in complete DMEM (Dulbecco's Modified Eagle Medium) medium supplemented with 10% FCS, 100 U/mL penicillin, 100 mg/mL streptomycin and 2 mM L-glutamine. Cells were routinely counted using trypan blue exclusion method.

### Mice and virus infections

M2stop.HY and M2MR.HY viruses were created using the galK mediated λRed recombineering as described previously [9], [68].The MHV68-H2bYFP BAC described in Collins et al [67] was used as a backbone to create a M2/galK intermediate BAC where the M2 ORF was replaced with a galK cassette. The M2/galK intermediate was made by amplifying the galK gene from pGalK using the following primers – gK-M2-FP (5′ tggagggggtttcaacaggcactagtctgatgaggtttcgttttcaggtaTCAGCACTGTCCTGCTCCTT-3′) and gK-M2-RP (5′-tccaggcgtgtttaaagaaaaagttatgttctgcgttagcaccttcactgCCTGTTGACAATTAATCATC GGCA-3′). These primers contain 50 bp homology arms that flank the M2 ORF. The resulting PCR product was transformed into the SW102/H2bYFP cells and screened by positive selection on minimal media containing galactose as the carbon source. Following confirmation of the M2/galK intermediate by restriction digest, the galK region was swapped out and replaced with either M2 or M2stop containing PCR products amplified from pMIT-M2 or pMIT-M2stop plasmids using the same homology arms used for generating the M2/galK intermediate: M2-FP (5′-tggagggggtttcaacaggcactagtctgatgaggtttcgttttcaggtaATGGCCCCAACACCCCCACA-3′) and M2-RP (5′- tccaggcgtgtttaaagaaaaagttatgttctgcgttagcaccttcactgTTATATATAGCGATAGGTAT CCTCCTCG – 3′). PCR products were electroporated into the M2/galK intermediate and recombinants were selected on minimal plates containing glycerol and 2-deoxy-D-galactose. Potential colonies were screened by colony PCR and confirmed by sequencing prior to final confirmation by Southern blotting (data not shown).

Eight-12 week old C57BL6/J mice (Jackson Labs) were used for harvesting splenocytes for retroviral transductions and for infections with M2stop.HY and M2MR.HY. IRF4^fl/fl^ mice have been previously described [29] and were purchased from Jackson Labs (catalog no. 009380). Mice were housed and maintained at the Whitehead vivarium according to Emory University and IACUC (International Animal Care and Use Committee) guidelines. Both male and female mice were used since we did not observe any significant gender specific differences in our preliminary latency or reactivation analyses. Mice were anesthetized using isoflourane before infecting with either 1000 PFU of MHV68-Cre or MHV68-WT virus (described in [8]) in 20 µLcomplete DMEM via the intranasal route.

### Limiting dilution analyses for assessing ex-vivo MHV68 reactivation and establishment of MHV68 latency

To determine the frequency of latently infected splenocytes, a limiting dilution, single-copy sensitive nested PCR assay was performed as previously described [5], [94], [95]. Briefly, frozen splenocytes were thawed, counted and washed in isotonic buffer prior to plating in serial three-fold dilutions onto a background of 10^4^ uninfected 3T12 cells in 96-well plates. Following lysis using a proteinase K mediated digestion step for 6–12 hours, the samples were subject to two rounds of nested PCR. Twelve PCRs were performed for each dilution for a total of six dilutions starting with 10^4^ cells. Each PCR plate contained control reactions that contained 0, 0.1,1, or 10 copies of plasmid DNA in a background of uninfected cells. Products were analyzed on a 2% agarose gel. To determine the frequency of splenocytes capable of reactivating virus from latency, a limiting dilution ex-vivo reactivation assay was performed as previously described [5], [94], [95]. Briefly, splenocytes from infected mice were plated in a two-fold serial dilution fashion (starting with 10^5^ splenocytes per well) on to MEF monolayers in 96-well tissue culture plates. Twenty-four wells were plated per dilution and 12 dilutions were plated per sample. Wells were scored microscopically for cytopathic effect (CPE) at 14–21 days post-explant. Preformed infectious virus was detected by plating parallel samples of mechanically disrupted cells onto MEF monolayers alongside intact cells.

### Immunoblotting and antibodies

Western blotting was performed by standard Lamelli protocol. Approximately 5×10^5^ cells per condition were harvested, washed once with PBS and resuspended in an appropriate volume of lysis buffer containing 50 mM Tris-HCl,150 mM NaCl,1 mM EDTA and 0.1% TritonX-100 supplemented with Complete mini-EDTA-free protease inhibitor tablet (Roche) and 1 mM NaF and 1 mM Na_3_VO_4_. The amount of protein in lysates was quantified using DC protein assay (Bio-Rad). For immunoblots using primary B cells, 10 µg protein was loaded per well. For cell lines, 5×10^5^ cell equivalents (unless when noted) were directly lysed in equivalent volumes of lysis buffer and 2× Lamelli buffer, boiled at 100°C for 5–10 minutes and resolved by SDS-PAGE. SDS-PAGE gels were transferred on to nitrocellulose membranes using a semi-dry apparatus (Bio-Rad).Blots were blocked with 5% milk in TBS-Tween (TBST) for one hour at room temperature followed by three washes in TBST. Primary antibodies were diluted in blocking buffer and incubated overnight at 4°C. Following overnight incubation, the blots were washed three times with TBST, incubated with secondary antibodies for one hour at room temperature followed by three washes in TBST. The blots were developed using Supersignal West Pico Chemiluminescent substrate (Pierce). Antibodies used include: goat anti-IRF4 (M17), NFATc1 (7A6), NFATc2 (4G6-G5) from Santa Cruz biotechnology (used at a 1∶200 dilution), rabbit polyclonal AU1 from Covance (1∶1000), mouse monoclonal β-Actin from Sigma (1∶10000). All secondary antibodies were from Jackson immunoresearch.

### Plasmids and luciferase constructs

pNFAT-luc, pNFkB-luc, pAP-1-luc and pFC-MEKK were obtained from (Agilent technologies) as part of the PathDetect Signal Transduction Pathway cis-reporting systems. pNFAT-luc contains 4× repeats of NFAT enhancer element sequence upstream of a luciferase reporter gene. pNF-kB-luc contains 5× repeats of the NFκB enhancer element upstream of a luciferase reporter gene. pAP-1 reporter plasmid contains 7× repeats of the AP-1 enhancer element upstream of a luciferase reporter gene. pISRE-luc reporter plasmid contains 5× repeats of an ISRE enhancer element upstream of a luciferase reporter gene. pFC-MEKK is a positive control expression plasmid that contains the activating MEKK kinase controlled by a constitutive CMV promoter. IκBαM is a negative control plasmid that expresses a mutant form of the IκB kinase.

The full length IL-10 promoter pGL2B-1538/+64, designated here as IL10pFL-luc, described in [61] was a courtesy of Dr.Stephen Smale, obtained from Addgene (Addgene plasmid 24942). The IL-10promoter constructs containing CNS9 and CNS3 elements, designated IL10pCNS3-luc and IL10pCNS9-luc were constructed by inserting the DNA fragments containing CNS3 or CNS9 into the full length IL-10promoter construct via MluI and XhoI as described in [60]. The DNA fragments containing CNS3 and CNS9 elements were PCR amplified from genomic DNA from naïve C57BL6 splenocytes. The primers used were CNS3 forward: 5′ GTGGTCATTTTTTCAGTAAGACC 3′, CNS3 reverse: 5′CCTAACCTTTCATC TCACAG 3′, CNS9 forward: 5′CGCTCTTCCGAATAGATGTGGT3′, CNS9 reverse: 5′CCGCACTCCGATGTATTAGTTCC3′. PCR products were cloned into pCR-BLUNT vector from Invitrogen to confirm sequence. Positive clones were PCR amplified, digested with MluI and Xho I and ligated into the full length promoter using MluI and XhoI sites. pMSCV-M2-IRES-Thy1.1(pMIT-M2) and pMSCV-M2stop-IRES-Thy1.1(pMIT-M2stop) are described in [16]. pMSCV-IRES-GFP-Empty vector (pMIG-EV) and pMSCV-IRF4-IRES-GFP (pMIG-IRF4) plasmids were kind gifts from Dr.X.Liang. pRV-NFAT1, pRV-NFAT2 and IL2promoter-luc described in [51], [66] were a courtesy of Dr.Anjana Rao, obtained from Addgene (Addgene plasmids 11100, 11101 and 12194 respectively).

### Transfections

1–1.5×10^6^ B cells were centrifuged, washed once with PBS and resuspended in 100 µL of Ingenio electroporation solution (MirusBio) or solution L (Lonza). Triplicate wells were used per condition. 5 µg reporter DNA prepared using Qiagen endofree maxiprep kit was added to the electroporation mixture and nucleofected using Amaxa Nucleofector I system. For M12 cells and the inducible cell clones derived from M12 as the parental cells, the program T-20 was used. To account for variability with doxycycline treated and untreated samples, two reactions worth of cells were pooled prior to nucleofection. Upon nucleofection, the pooled reactions were split into two, one receiving doxycycline while the other was left untreated. Reporter activity was measured after 48 hours as per manufacturer's instructions (Promega luciferase assay system).

### Retrovirus production and transduction of primary B cells

Splenocytes were harvested from 9–12 week old C57BL6 mice (Jackson Labs), and primary B cells were isolated by immunomagnetic negative selection using the EasySep Mouse B Cell Enrichment Kit (Stem Cell Technologies) as per manufacturer's instructions. The purity of B cell isolation was routinely analyzed and found to be ≥95% as determined by staining for CD19 by flow cytometry. The cells were plated at 1×10^6^ cells/mL of a 24 well plate overnight with cRPMI containing 25 µg/mL LPS (Sigma) prior to retroviral transduction. Retroviruses were prepared as described in [16]. Briefly, BOSC23 (ATCC) producer cells were plated at a density of 1.5×10^6^ cells per 60 mM collagen coated dish (BD biosciences) overnight. After 18–24 hours of plating, the cells were transfected with 5 µg of either pMIT-M2 or pMIT-M2stop. Supernatants were harvested at 72 hours and used immediately or frozen at −80°C until ready for use. On the day of transduction, the retroviruses were centrifuged at 2000rpm for 10 minutes to remove any cellular debris and supplemented with 5 µg/mL of polybrene. 750 µL of the media was removed and replaced with 1 mL of the retrovirus containing polybrene. The cells were spin infected for 2500 rpm for one hour at 30°C. Post-transduction, 750 µL of the supernatant was removed from each well and 1 mL of fresh media was added. Triplicate wells per condition were analyzed by flow cytometry at days 2–5 post-transduction.

### Flow cytometry

Flow cytometry was performed as per standard procedure. Briefly, cells from triplicate wells per condition were washed once in FACS buffer (2% FBS in PBS) and incubated with Fc block (Rat anti-mouse CD16/CD32, eBioscience) for 10 minutes prior to surface staining with an antibody cocktail. The cells were washed two times with FACS buffer prior to analysis on a BD LSRII flow cytometer. Antibodies used include: CD19-FITC, - APC, - PE, -PECy7 or -APC-Cy7, Thy1.1-APC, GL7-FITC, CD23-PE, IgD-FITC, B220-PECy7, CD138-APC, IA^b^-PE (eBioscience or BD biosciences). Live dead analysis was routinely included using LIVE/DEAD® Fixable Violet Dead Cell Stain Kit (Invitrogen).

Intracellular staining for IRF4 was performed as described in [28] with minor modifications. Briefly, 5×10^6^ cells were surface stained for surface markers as described above. Live/dead analysis was included using Zombie Yellow fixable viability stain (Biolegend). After surface staining, cells were fixed with 1%(w/v) paraformaldehyde (Electron Microscopy Sciences) followed by permeabilization with 1×(0.05–0.15%) stock made from 10× saponin-based permeabilization and wash buffer(Invitrogen), diluted in FACS buffer. Cells were incubated overnight in 1× saponin buffer with an antibody cocktail containing a rabbit anti-GFP-FITC antibody at a dilution of 1∶500 (Rockland Antibodies) and a polyclonal goat anti-IRF4 at a dilution of 1∶100 (Santa Cruz Biotechnology) in the presence of 5% (v/v) normal donkey serum. Normal goat IgG (Santa Cruz Biotechnology) was used as an isotype control. Cells were washed two times with 0.5× saponin based wash buffer followed by incubation with an Alexa-647-coupled donkey anti-goat detection antibody (Jackson Immunoresearch). Cells were washed two times prior to analysis. Data was analyzed using FlowJo software (TreeStar, Inc.).

### Drug treatments

Cyclosporin A (500 ng/mL), FK-506 (1 µM), EGTA (2.5 mM), PMA (60 ng/mL) and Ionomycin (1 mM) were from Sigma. BAPTA-AM (5 µM), PP2 (10 µM) and PP3 (10 µM) were from Calbiochem. The selection drugs, hygromycin and G418 were from Calbiochem and Cellgro, respectively. Doxycycline (5–500 ng/mL) was from Clontech. The drugs were used at a final concentration as indicated within parentheses above. Cell viability upon drug treatment was assessed by trypan blue exclusion.

### ELISA

ELISA for IL-10 was performed as per manufacturer's instructions (BD OptEIA™, BD Biosciences). Supernatants from cells were stored at −80°C prior to ELISA analysis.

### Statistical analysis

Statistical data analysis was performed using GraphPad Prism software. Data shown represents one of at least triplicate experiments. Error bars represent standard error mean. Significance was determined by two-tailed, unpaired Student's t-test with a confidence level of 95%.

## Supporting Information

Figure S1 **M2 expression activates the IL2promoter.** 3×10^6^ DS10 cells were nucleofected with 10 µg of the IL2-promoter-luc plasmid using Ingenio solution as described in Figure 2A. After transfection, the cells were divided into two reactions - one half was left untreated and the other half received doxycycline for 48 hours prior to quantitating luciferase activity. Each condition was performed in triplicate wells. Luciferase activity is represented as fold over uninduced ± SEM. In reactions with CsA addition, CsA was added at 24 hours post-nucleofection. Treatment with PMA and Ionomycin served as a positive control.
(TIF)

Figure S2 **Addition of drugs to DS10 cells does not have a significant effect on cell viability.** (A–C) DS10 cells were used to perform a NFAT-reporter assay as in Figure 3A–3C. (D) Cells from panel A–C were counted using trypan blue exclusion to determine the effect of the drugs on the viability of the cells. Viability was plotted as a percentage of uninduced, setting uninduced samples to a 100%. (E) Live cell numbers were plotted as fold over uninduced, setting the uninduced samples to 1. Data is representative of an average of counts from three replicate wells per condition.
(TIF)

Figure S3 **Effect of drugs on levels of M2 and IRF4 expression.** (A and C) Replicate wells of DS10 cells were treated with drugs as in figure S2A–2C. Whole cell lysates were harvested and 40 µg of protein was analyzed by western blotting for levels of M2 expression (using an AU1 antibody) and IRF4 expression. (B) Supernatants from figure S3A were analyzed for IL10 levels by ELISA. Data is representative of duplicate wells per condition.
(TIF)

Figure S4 **IL10p-CNS9-luc has the maximal activity upon M2 expression.** IL-10pFL-luc, IL10pCNS-3-luc and IL10pCNS-9-luc plasmids (described in Materials and Methods) were nucleofected into DS10 cells as described in Figure 6C. Luciferase activity is plotted as fold over uninduced controls.
(TIF)
