# Supplementary figures and images for: Murine Gammaherpesvirus M2 Protein Induction of IRF4 via the NFAT Pathway Leads to IL-10 Expression in B Cells
Source: PLoS Pathog. 2014 Jan 2;10(1):e1003858. doi: 10.1371/journal.ppat.1003858 (PMC3879372; doi:10.1371/journal.ppat.1003858)

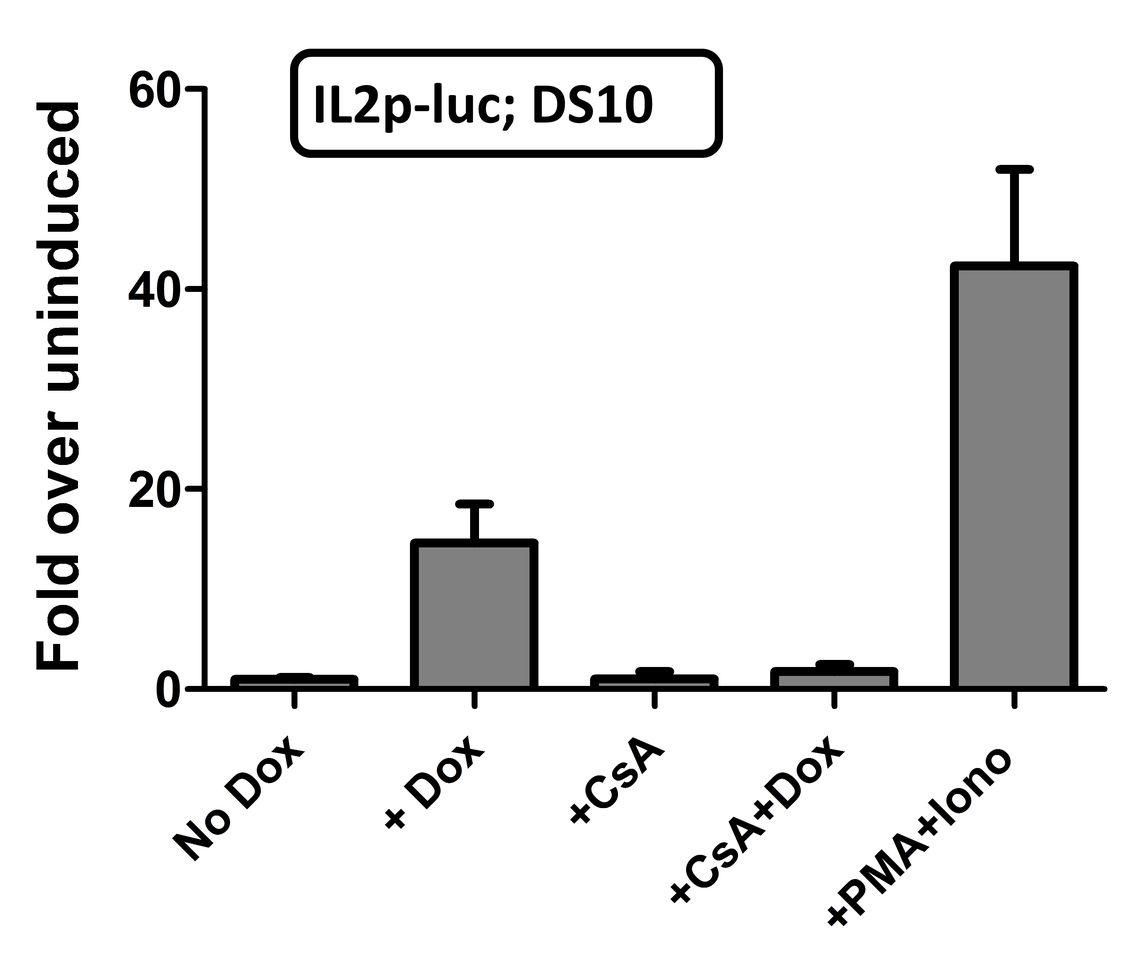

Supplement: Figure S1 — M2 expression activates the IL2promoter. 3×10^6^ DS10 cells were nucleofected with 10 µg of the IL2-promoter-luc plasmid using Ingenio solution as described in Figure 2A. After transfection, the cells were divided into two reactions - one half was left untreated and the other half received doxycycline for 48 hours prior to quantitating luciferase activity. Each condition was performed in triplicate wells. Luciferase activity is represented as fold over uninduced ± SEM. In reactions with CsA addition, CsA was added at 24 hours post-nucleofection. Treatment with PMA and Ionomycin served as a positive control. (TIF) [file ppat.1003858.s001.tif]

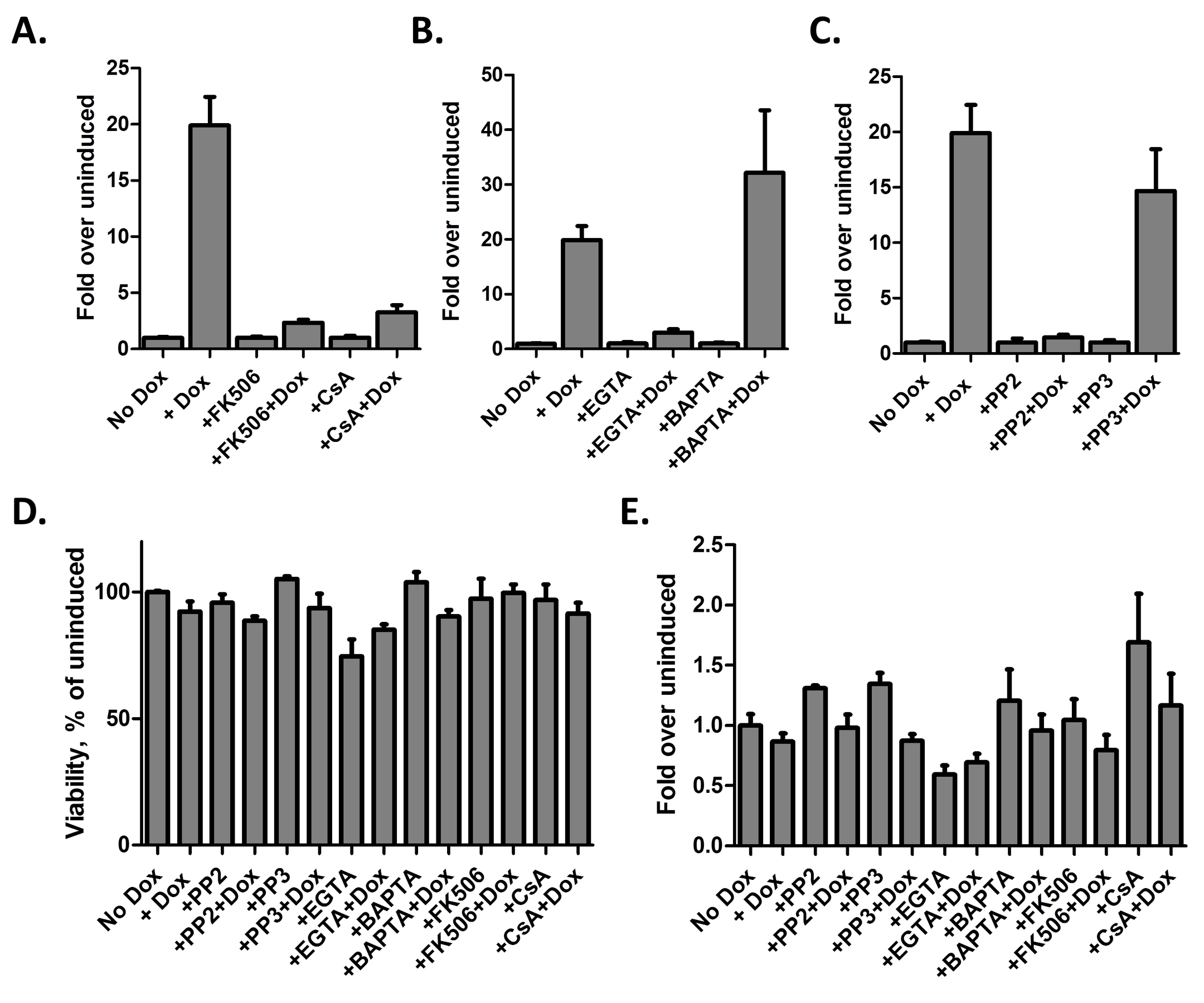

Supplement: Figure S2 — Addition of drugs to DS10 cells does not have a significant effect on cell viability. (A–C) DS10 cells were used to perform a NFAT-reporter assay as in Figure 3A–3C. (D) Cells from panel A–C were counted using trypan blue exclusion to determine the effect of the drugs on the viability of the cells. Viability was plotted as a percentage of uninduced, setting uninduced samples to a 100%. (E) Live cell numbers were plotted as fold over uninduced, setting the uninduced samples to 1. Data is representative of an average of counts from three replicate wells per condition. (TIF) [file ppat.1003858.s002.tif]

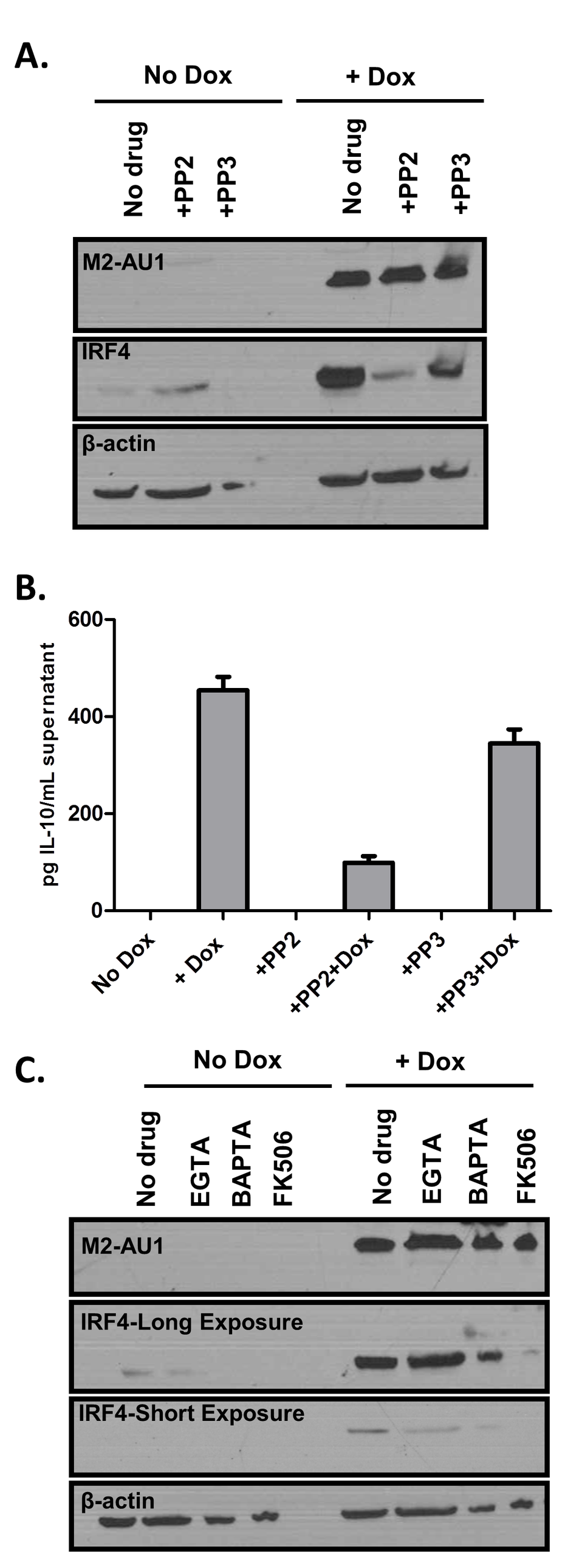

Supplement: Figure S3 — Effect of drugs on levels of M2 and IRF4 expression. (A and C) Replicate wells of DS10 cells were treated with drugs as in figure S2A–2C. Whole cell lysates were harvested and 40 µg of protein was analyzed by western blotting for levels of M2 expression (using an AU1 antibody) and IRF4 expression. (B) Supernatants from figure S3A were analyzed for IL10 levels by ELISA. Data is representative of duplicate wells per condition. (TIF) [file ppat.1003858.s003.tif]

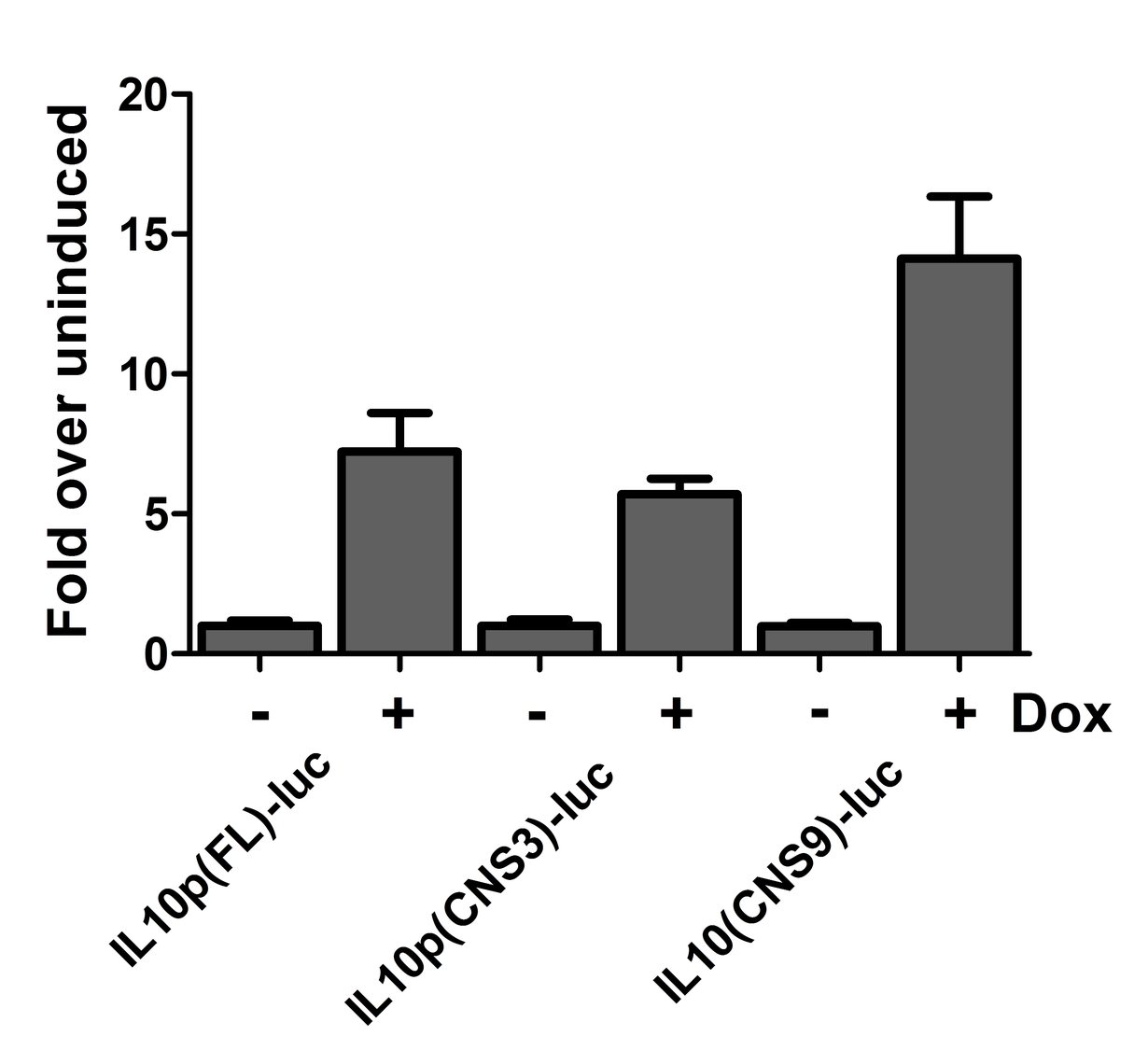

Supplement: Figure S4 — IL10p-CNS9-luc has the maximal activity upon M2 expression. IL-10pFL-luc, IL10pCNS-3-luc and IL10pCNS-9-luc plasmids (described in Materials and Methods) were nucleofected into DS10 cells as described in Figure 6C. Luciferase activity is plotted as fold over uninduced controls. (TIF) [file ppat.1003858.s004.tif]
